# Building bridges of excellence: a comprehensive competence framework for nurses in hospice and palliative care—a mixed method study

**DOI:** 10.1186/s12904-023-01318-x

**Published:** 2023-12-12

**Authors:** Wei-Ying Li, Ying Fang, Yi-qing Liang, Shu-qin Zhu, Ling Yuan, Qin Xu, Yue Li, Yin-long Chen, Chang-xian Sun, Xiao-xu Zhi, Xiao-yan Li, Rong Zhou, Mai Du

**Affiliations:** 1 School of Nursing, Nanjing Medical University, Nanjing, 211166 P. R. China; 2 School of Medicine, Jiangsu University, Zhenjiang, 212000 China; 3 The Comprehensive Cancer Centre of Drum Tower Hospital, Medical School of Nanjing University, Clinical Cancer Institute of Nanjing University, Nanjing, 210008 P. R. China; 4 Jiangsu Institute of Quality and Standardization, Nanjing, 210029 China; 5 School of Health Sciences, Jiangsu Vocational Institute of Commerce, Nanjing, 211168 China; 6 Nursing Department, Jiangsu Cancer Hospital and Nanjing Medical University Affiliated Cancer Hospital and Jiangsu Institute of Cancer Research, Nanjing, 210009 China; 7 Hospice Unit, The Air Force Hospital From Eastern Theater of PLA, Nanjing, 210002 China

**Keywords:** Hospice and palliative care nursing, Competence, Systematic review, Delphi technique, Interview, Cross-sectional study

## Abstract

**Background:**

Hospice and Palliative Care (HPC) is in high demand in China; however, the country is facing the shortage of qualified HPC nurses. A well-suited competence framework is needed to promote HPC human resource development. Nevertheless, existing unstandardized single-structured frameworks may not be sufficient to meet this need. This study aimed at constructing a comprehensive multi-structured HPC competence framework for nurses.

**Methods:**

This study employed a mixed-method approach, including a systematic review and qualitative interview for HPC competence profile extraction, a two-round Delphi survey to determine the competences for the framework, and a cross-sectional study for framework structure exploration. The competence profiles were extracted from publications from academic databases and interviews recruiting nurses working in the HPC field. The research team synthesized profiles and transferred them to competences utilizing existing competence dictionaries. These synthesized competences were then subjected to Delphi expert panels to determine the framework elements. The study analyzed theoretical structure of the framework through exploratory factor analysis (EFA) based on a cross-sectional study receiving 491 valid questionnaires.

**Results:**

The systematic review involved 30 publications from 10 countries between 1995 and 2021, while 13 nurses from three hospitals were interviewed. In total, 87 and 48 competence profiles were respectively extracted from systematic review and interview and later synthesized into 32 competences. After the Delphi survey, 25 competences were incorporated into the HPC competence framework for nurses. The EFA found a two-factor structure, with factor 1 comprising 18 competences namely Basic Competences; factor 2 concluding 7 competences namely Developmental Competences.

**Conclusions:**

The two-factor HPC competence framework provided valuable insights into the need and directions of Chinese HPC nurses’ development.

**Supplementary Information:**

The online version contains supplementary material available at 10.1186/s12904-023-01318-x.

## Background

In mainland China, there is a high demand for hospice & palliative care (HPC) due to the ageing population, rising burden of chronic diseases, and poor quality of death. China’s seventh national census of 2020 reported the number of individuals aged ≥ 65 years was 190.64 million, accounting for 13.50% of the total population [1]. Over 75.8% of the Chinese older adults had ≥ 1 chronic disease, [2] and the deaths due to chronic diseases accounted for 88.5% of annual total deaths [3]. This indicates that each year, a significant number of Chinese older adults require HPC, and the need would increase further when other age groups of end-of-life patients are added. A cross-sectional study, published in The Lancet, utilized comprehensive databases such as the National Center for Pediatric Cancer Surveillance, the nationwide Hospital Quality Monitoring System, and public databases covering over 31 provinces in mainland China. This study aimed to estimate the incidence of cancer among children (aged 0–14 years) and adolescents (aged 15–19 years) in China, revealing that 121,145 cancer cases were diagnosed between 2018 and 2020 [4]. This significant figure underscores a substantial demand for pediatric HPC in China. A retrospective study reflecting on a six-year experience in the development of a pediatric palliative care service in a tertiary children's hospital in China highlighted the feasibility and positive impact of palliative care services, particularly counseling services, on children with end-of-life illnesses. However, the study emphasized the persistence of unmet pediatric palliative care needs, warranting ongoing attention [5]. Beyond the evident demand for HPC, China also needs to improve the quality of death, as it was ranked 53rd out of 81 nations and territories in the Quality of Death and Dying 2021 report [6]. These situations highlight the urgent need for comprehensive and improved HPC services in China.

However, the HPC development in China is facing various challenges, such as the traditional concept of death, insufficient financial support, and a shortage of HPC professionals, including HPC nurses. A notable number of the Chinese population is profoundly influenced by Confucianism, which places high importance on “filial piety” [7–9]. This cultural aspect may make it challenging for individuals to decide to forgo non-essential resuscitation for their parents or other elders. Because individuals unfamiliar with the concept of HPC may equate it with passive acceptance of impending death, contrary to their deeply held beliefs [10]. Consequently, Chinese HPC professionals confront unique cultural challenges, necessitating them to undertake the responsibility of educating and disseminating the principles of HPC. Predominantly, this responsibility has been shouldered by HPC nurses.

Simultaneously, certain HPC services, such as psychological support, spiritual care, and bereavement care, remain excluded from the hospital’s billing system. As a result, HPC professionals, especially nurses, are compelled to offer these essential services on a pro bono basis to patients and caregivers. The immature HPC charging model has been a subject of ongoing discussion within the development of HPC in China, yet a definitive solution remains elusive. Unlike well-established HPC systems where multidisciplinary teams collaborate seamlessly, in China, HPC is predominantly undertaken by doctors and nurses alone, [11] placing a substantial burden on HPC nurses in terms of workload and diverse responsibilities. Consequently, the challenges highlighted in this paragraph cast a shadow on the human resource development of HPC nurses.

The government has taken actions to overcome above obstacles, such as incorporating HPC into national health planning, announcing HPC pilot cities, and issuing national HPC practice guidelines and management standards [12]. These policies and approaches have promoted the establishment of independent HPC institutions and HPC departments or HPC wards at different levels of hospitals. However, there has been an increasing disparity between the growing social needs for HPC and the availability of well-trained doctors and nurses in this field. In the current stage, the direct impact of these policies on alleviating the shortage of HPC professionals, including HPC nurse, was not significant. Establishing a robust training system for HPC, enabling general nurses to acquire essential HPC competencies, and facilitating specialized HPC nurses' continuous improvement in their capabilities could potentially address the challenges we currently face.

Domestic medical school has not established HPC undergraduate program. Chinese nurses learn HPC knowledge and skills mainly through continuous education. But the available HPC continuous education has several limitations. For example, programs designed and provided by different hospitals or associations lead to the absence of standardization on knowledge content, training strategy, and evaluation criteria. Programs were primarily one-time, leading to an unclear pathway for the self-growth and development of HPC nurses. A lack of available nurses’ HPC competence assessment tool meant nurses are unable to identify their most suitable program and study objectives. Although, national level associations, such as the Chinese Nursing Association, provided well-organized HPC continuous education programs, they can only take a limited number of participants. These issues may harm the motivation for nurses working in the HPC field. Therefore, it is crucial to develop a rigorous training and evaluation system which with no doubt requires a well-established HPC competence framework as foundation.

Scholars and some overseas associations have published HPC competence frameworks for nursing students, general nurses, and HPC specialized nurses [13–15]. Due to the differences of socio-economic backgrounds, framework development time, culture traditions, and nurses’ characteristics, the competences listed in each formwork were differ. This fact creates the obstacles when using existing frameworks. Furthermore, previous competence frameworks were mono-structured, which may not fulfil the need of continuous development. Given the uneven global development HPC, [16] we should adopt an international perspective to design and establish a comprehensive and dynamic HPC competence framework to provide a theoretical basis for the assessment, training, and management of HPC competence for scholars from regions at different stages of development.

The objectives of the study are 1) to comprehensively integrate HPC competence profiles for nurses from published research articles and other reliable publications, 2) construct the HPC competence framework for nurses, and 3) explore the underlying structure of the constructed framework.

## Method

**Fig. 1:**
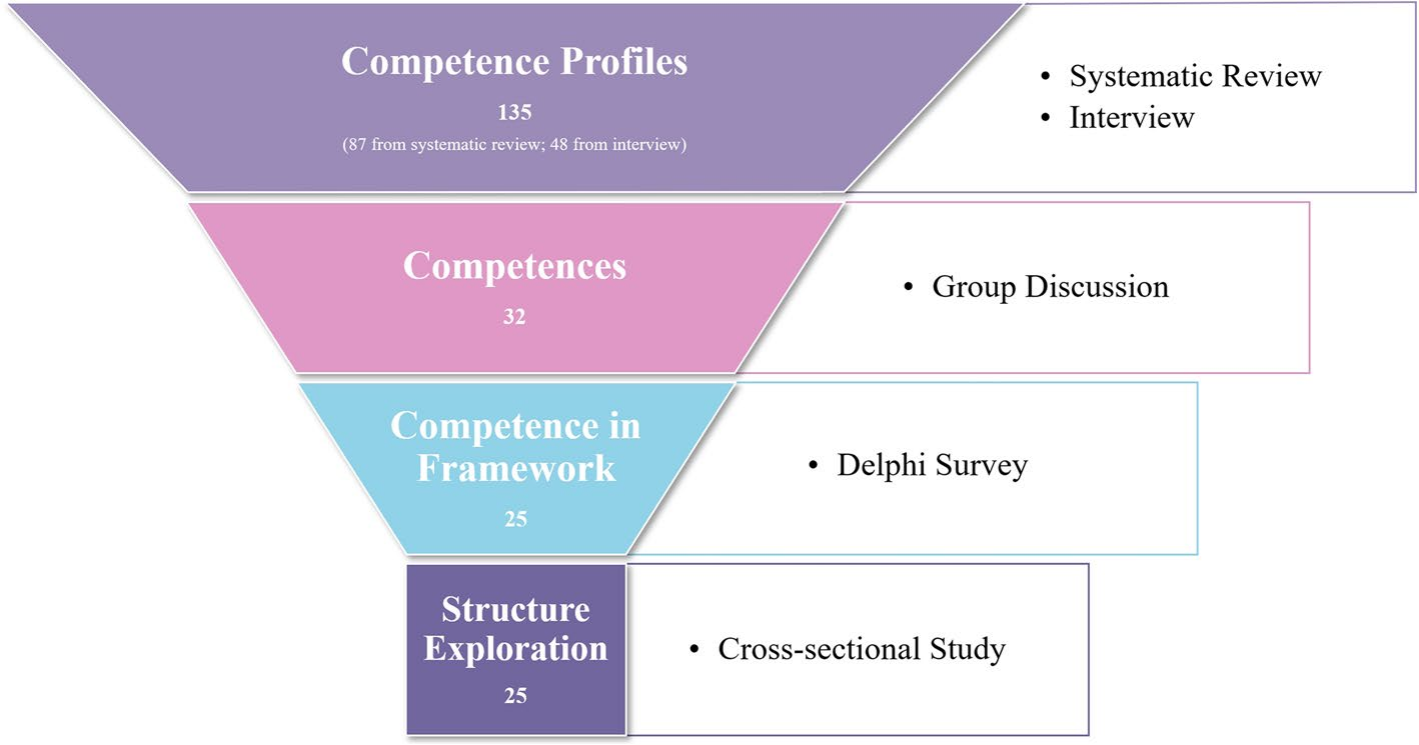
Study steps and number of corresponding competences

This study was designed in four steps using a mixed-method approach (Fig. 1). The ethical approval was obtained from the Ethics Review Committee of the Nanjing Medical University (NMU2020-277), all steps recruited participants obtained written or online informed consent.

### Step 1-competence profiles extraction

This step involved extracting competence profiles for HPC nurses through a systematic review of literature and a qualitative interview with clinical nurses working in the HPC field, using the Behavioral Event Interview (BEI) framework.

**Fig. 2:**
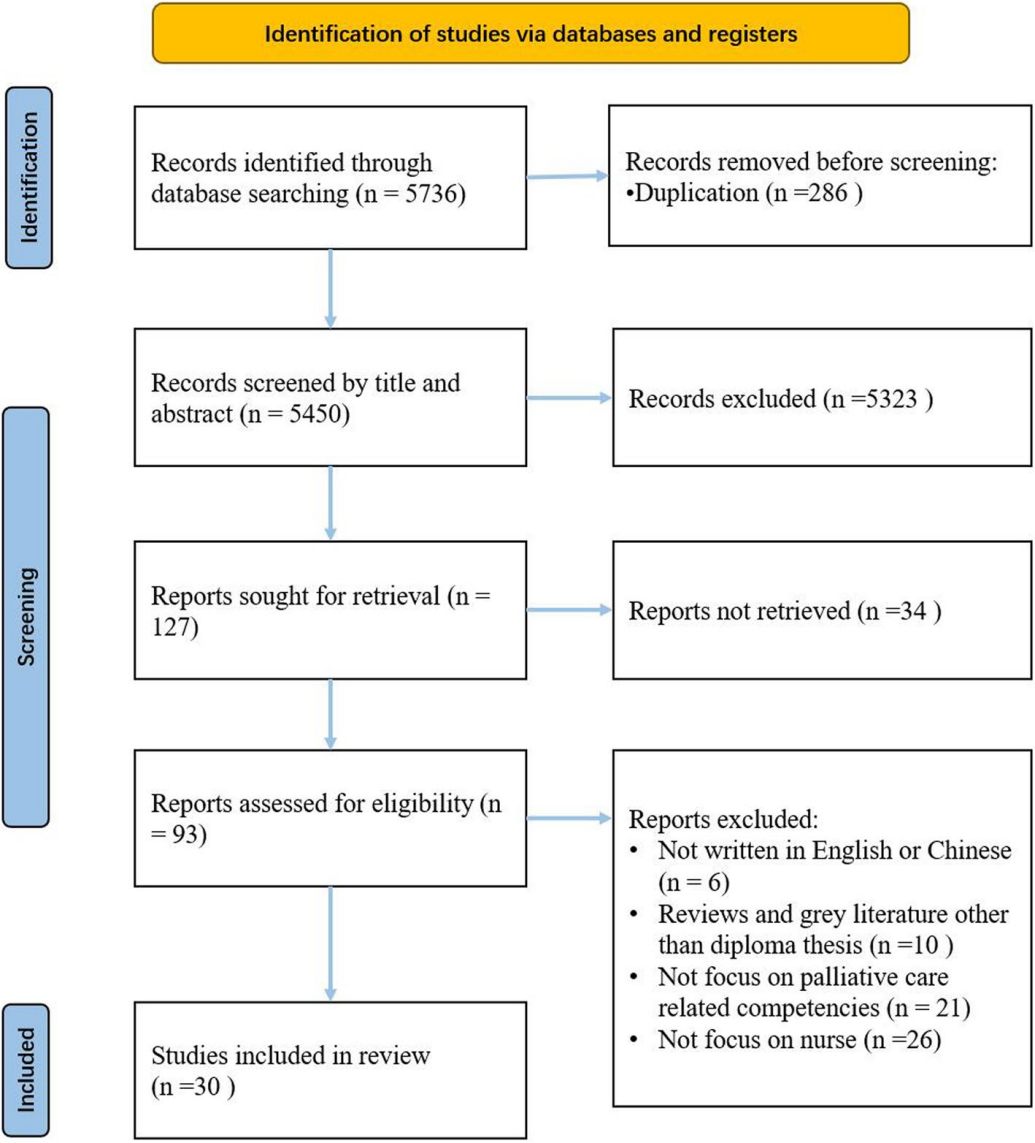
Systematic review PRISMA diagram

The systematic review followed PRISMA guideline [17]. We used Endnote X9 to manage and select the studies. Two authors independently conducted two rounds of systematic searches across six databases, with the first round completed in 2020 and the second in March 2022. The search focused on keyword themes: palliative and hospice care, competence, and nurse. Additional file 1 provides detailed information about the keywords, search strategy, and literature selection criteria. From a total of 5736 identified publications, 30 were included for data extraction and narrative synthesis, as shown in Fig. 2 [13, 14, 18–45]. The information extracted from each study included author, year, country, article type/ study design, target nurse, and ability/ competence profiles and corresponding context (if any). The authors integrated the extracted competence profiles by referring to known competence dictionaries [46–48].

The BEI framework, which involves asking candidates to describe their self-evaluated successes and failures on the job, is widely used for competence extraction and summary [48, 49]. Thus, we used this framework to design the interview outline (Additional file 2) and employed the STAR strategy, which prompts interviewees to describe the specific ‘situation, task, action, and result’ of their successes and failures [48].

Our sampling strategy was guided by the concept of “saturation”, where the sample size is determined once no new themes emerge [50]. Diversity in the sample was sought by recruiting participants across a variety of hospitals, working department, and years of HPC experience. Eventually, 13 interviewees who met the selection criteria (Table 1) were recruited through purposeful sampling.

**Table 1:** Participants’ selection criteria of qualitative interview, Delphi survey, and online cross-sectional study

Study	Inclusion criteria	Exclusion criteria
Qualitative interview	1. Working in oncology department or hospice and palliative ward or working in the ward that having hospice and palliative care bed 2. ≥ 1 year experience of taking care of end-of-life patients 3. Recommending by their nurse managers	1. Not willing to participant the interview
Delphi survey	1. From hospice pilot cities or being the member of Chinese Nursing Association Hospice Professional Committee 2. Having bachelor diploma or above 3. Above 5-year working experience as the nurses, doctors, or research fellows engaging in HPC clinical management or research	1. Not response or reject the invitation
Online cross-sectional study	1. Nurse working in hospitals in HPC pilot cities 2. Either taking care of end-of-life patients in the past 12-month or have received the HPC training	1. Not willing to participate the survey

Before formal interviews, the corresponding author established a familiar relationship with potential interviewees through clinical field research and observation. This work laying the foundation for in-depth interviews. The 60-min face-to-face interview was conducted in the quiet and undisturbed spaces in the participants working department. The corresponding author conducted and recorded each interview, which was later transcribed.

Following each interview, the verbatim transcripts were uploaded to NVivo 11 for Windows by one author other than the corresponding author and analyzed using a thematic approach, with three authors independently generating initial codes line by line [51]. The relevance of codes was documented during analysis. Peer debriefing within our research team occurred fortnightly at the initial coding stages to discuss varying interpretations of emerging codes and ensure the analysis captured the full range and depth of the data. Subsequent discussions involved a wider group of researchers and clinical HPC experts. Once a comprehensive set of codes was established across the dataset, they were organized into themes based on pre-existing competence dictionaries [46, 48].

### Step 2-competence synthesis

Furthermore, a group discussion was held with five HPC experts where the research team integrated all the competence profiles obtained from the systematic review and the interview. The corresponding competence of each profile was identified according to the definitions given by known competence dictionaries [46–48]. After the identification process, any duplicate competences were eliminated, and synonymous ones were merged.

### Step 3-competence in framework confirmation

We used a two-round Delphi survey to determine the competences included in the framework. Delphi surveys involve multiple rounds of anonymous questionnaires completed by a panel of experts and are commonly used for developing competence frameworks in healthcare [52].

Guided by the principle of purposefulness, a comprehensive selection process was employed, [53] taking into account various aspects such as professional expertise, years of HPC work experience, job position, and educational background. Our aim was to identify national-level medical experts, nursing managers, and researchers actively engaged in the field of HPC. Preference was given to experts with substantial experience in HPC, possessing extensive clinical expertise or a notable research background in this specialized area. To ensure a representative panel, our selection process focused primarily on experts working in HPC pilot cities and members of the Chinese Nursing Association Hospice Professional Committee. We planned to recruit 15 to 20 experts and the specific expert panel selection criteria is available in Table 1 [53, 54].

We designed a questionnaire that included competences synthesized from last step with definitions and sent it to all experts via email. The importance of each competence was rated by a 5-point Likert scale ranging from 1 (“not important at all”) to 5 (“extremely important”) in both rounds, and experts were also asked to propose comments or suggestions for each competence and recommend adding additional competences to the framework. We assessed the reliability and validity of the Delphi survey using expert opinion consensus and calculated the positive coefficient, authority coefficient (Cr), and coordination coefficients (Kendall’s Concordance Coefficient, ω). The Cr is defined as $Cr = (\mathrm{familiarity} + \mathrm{criterion})/2$. We determined the retention items based on the mean, standard deviation (SD), and coefficient of variation (CV) of each competency's importance score and set the retention threshold at a mean < 3 or CV > 0.25.

### Step 4-theoretical structure exploration

To explore the theoretical structure of the competence framework, we conducted an online cross-sectional survey sending an online questionnaire to domestic nurses in the HPC field working in different hospitals in 10 pilot cities. The nurses reported socio-demographic information, such as gender, marital status, educational level, and workplace characteristics. Additionally, they rated the importance of the competences using the same 5-point Likert scale as Delphi survey. To ensure to meet the suggested valid sample size of factor analysis (n > 300), [55, 56] we planned collecting at least 600 online questionnaires by convenience sampling.

We excluded questionnaires that did not meet certain criteria, including completion time less than 150 s, completed by nurses who did not care for end-of-life patients or receive HPC training, did not sign the e-informed-consent, or showed below-expected respondent focus. Descriptive statistics was used to summarize the characteristics of nurses and calculate the mean, SD, CV of the importance score. The exploratory factor analysis was employed to explore the underline factor structure. The Kaiser–Meyer–Olkin (KMO) Measure of Sampling Adequacy and Bartlett’s Test of Sphericity were first conducted to assess the suitability of the data for factor analysis [57]. Exploratory factor analysis (EFA), principal component analysis, was first performed unrotated, using maximum likelihood extraction and eigenvalues > 1. Additionally, we performed EFA with varimax rotation. SPSS (version 27.0) was used for all analyses.

## Results

### Competence extraction and synthesis

Twenty-five research articles [13, 14, 18–20, 22, 23, 26–43] and five national frameworks [21, 24, 25, 44, 45] published between 1995 and 2021 from China (*n* = 11), [28–33, 37, 38, 42, 43] America (*n* = 5), [18–20, 23, 25] Canada (*n* = 3), [13, 21, 22] Finland (*n* = 3), [35, 36, 40] Korea (*n* = 1), [14] Ireland (*n* = 1), [41] Iran (*n* = 1), [27] Italy (*n* = 1), [34] Australia (*n* = 1), [45] the United Kingdom (*n* = 1), [44] New Zealand (*n* = 1), [24] Germany (*n* = 1) [26] were included in the synthesis (Additional file 3). The publications presented the HPC competences for different nurses, such as palliative care nurse [19, 22, 29, 35, 43, 45] [also described as palliative care Advanced Practice Nurse (APN), nurses in palliative care, nurses in primary care settings, specialist palliative care nurses; *n* = 6], hospice nurses [18, 20, 28, 31, 37, 39, 42] (also described as hospice nursing specialist nurses, hospice care specialized nurses; *n* = 7), oncology nurses [27, 30, 31, 38] (also described as health professionals involved in cancer care, *n* = 4), HPC nurses [14, 21, 23, 32, 34] (also described as HPC specialized nurses, *n* = 5), and general RN [13, 24–26, 40, 41] (also described as nurses, nurses with undergraduate diploma, undergraduate level nurses; *n* = 6). Two articles [36, 44] studied the HPC competences of both generalist palliative care nurses and specialty palliative care nurses. After removing duplicates, the systematic review extracted a total of 87 competence profiles (Table 2).

**Table 2:** Competence profiles extracted from systematic review and qualitative interview

Extracted from systematic review		Extracted from qualitative interview	
1	resource management ability	1	grief counseling
2	disease management ability	2	basic care
3	confidence	3	home care
4	multidisciplinary cooperation	4	spiritual care
5	evaluating capability	5	symptom assessment
6	information acquisition and processing	6	humanistic care
7	communication ability	7	social support
8	perseverance	8	promote physical comfort
9	facilitate decision-making ability	9	everyday life care
10	judgment ability	10	values
11	creativity	11	love
12	leadership	12	gratitude
13	influence	13	patience
14	support	14	affinity
15	reflection ability	15	kindness
16	evidence-based awareness	16	empathy
17	risk assessment	17	talent
18	objectivity	18	communication and coordination skill
19	audit	19	innovation
20	responsibility	20	reflection ability
21	commitment	21	observation ability
22	efficient use of resources	22	role change
23	innovation	23	leadership
24	recognize the importance of multidisciplinary collaboration	24	emotion management
25	recognize the impact of decision making on patients	25	adaptive ability
26	understanding	26	psychological regulation ability
27	responsiveness	27	study ability
28	proactiveness	28	death education
29	understanding informed principles	29	mental comfort
30	understanding the principles of independent decision-making	30	information support
31	awareness of consultation	31	nutritional management
32	empowerment	32	sensitivity
33	promote the patient-nurse relationship	33	service awareness
34	provide information and resources	34	hospice philosophy
35	research ability	35	encouragement
36	evidence-based practice competency	36	proactivity
37	identification ability	37	train
38	inquisitiveness	38	cultivate others
39	energetic	39	gain the trust of patients
40	educational ability	40	respect patients
41	self-awareness	41	growth
42	knowledgeable	42	stress management
43	teaching demonstration ability	43	courage
44	creating a learning environment	44	responsibility
45	coordination ability	45	professional dedication
46	overall viewpoint	46	good at mobilizing resources outside of nursing
47	study ability	47	team building
48	promote professional development	48	self-motivation
49	organizational ability		
50	critical thinking		
51	self-evaluation		
52	fairness		
53	achievement orientation		
54	sensitivity		
55	reflection ability		
56	display		
57	collaboration ability		
58	recognize the need to grieve		
59	listening		
60	respond to the needs of bereaved parents		
61	respect		
62	distinguish between normal grief and abnormal grief		
63	boundary awareness		
64	provide consultation		
65	transformation		
66	understand performance appraisal and personal development		
67	interpersonal ability		
68	service spirit		
69	analytical ability		
70	create a good environment		
71	dealing with emotions		
72	draft a plan		
73	strain ability		
74	flexibility		
75	assessment and management of pain		
76	symptom management and prognosis		
77	intervention ability		
78	bereavement care		
79	interprofessional cooperation		
80	understand ethics		
81	enterprise		
82	expression ability		
83	empathy		
84	anticipatory		
85	encouragement		
86	focus on specific populations		
87	participate in quality improvement		

The characteristics of the interviewed nurses were as Table 3. We presented an example of the thematic analysis in Table 4. In total, 48 competence profiles were extracted from the interview (Table 2). After competence identification, duplicates removal, and synonym combination, 32 competences were synthesized by the research group.

**Table 3:** The characteristic of the 13 interviewed female nurses

No	Age	Education level	Marriage	Department^a^	Years of HPC experience	Transcripts word count
1	31	Master	Unmarried	Oncology	1	12,585
2	40	Bachler	Married	Pain management	10	7164
3	29	Bachler	Unmarried	Oncology	3	16,486
4	29	Bachler	Unmarried	Oncology	5	7640
5	36	Bachler	Married	Oncology	5	6578
6	34	Bachler	Married	Oncology	6	4662
7	27	Short Cycle	Married	HPC	5	3985
8	30	College	Married	HPC	5	11,831
9	23	College	Unmarried	HPC	3	4482
10	27	College	Unmarried	HPC	4	7214
11	27	Bachler	Unmarried	HPC	3	7173
12	23	Bachler	Unmarried	HPC	1	4104
13	25	College	Married	HPC	4	7571

^a^The HPC departments were joint with internal medicine department or admitting patients of internal medicine department

**Table 4:** Example of thematic analysis of interviews

Transcripts	Theme code	Competence profile
…… a patient is about 13 to 14 years old. In this age he already has the concept of live and death. By the time he came to us, we could obviously feel that he was very sullen, his eyes were very empty. I am not saying that he has no ability of communication. I think he is maybe, more like being afraid, scared or powerless. He maintained this situation every day. His parents, young parents, don't know what to do, they don't know if it's the right decision taking the child to here (HPC ward), after all their child is still a kid Although this child is young now, he has concept about live and death. He does not know where he will go if he really dies one day. I told the father that he could give his child more love and attention. The father said, “I want to kiss him, or touch him, but he is very reluctant, he gave me not respond.” I explained to the father that his child now scared and full of fear. Whether the child gives a reaction or not, you have to express your love for him. Like no matter where he goes, even if he goes to another world, you have to let him feel the warmth of family, parental care, to give him this feeling of love ……	• sensitively detect the child-patient’s abnormality and be able to analysis the possible problem • understand the difference of perceptions and behaviors of young parents and their child due to different backgrounds • guide parents to respond positively to their child's psychological problem • encourage the father when he wanted to back down	Sensitivity Observation ability Empathy Information support Encouragement

The original transcript and theme codes were Chinese, one author translated the sample paragraphs to English only for this paper

### The competence confirmation

For Delphi study, 16 experts (2 males, 14 females) who have on average 14.38-year (ranging from 7 to 34 years) HPC experience accepted the invitation. The panel included a doctor and four nurses with middle-level professional titles; one doctor, two research fellows, and five nurses with associate senior-level professional titles; three nurses with the senior-level professional title. The positive coefficient measured by the response rates were 100% and 81.25% (13 out of 16) in two-rounds indicating high positive coefficient [58]. In the first round, the Cr was 0.87 with 0.80 familiarity and 0.94 criterion; in the second round the Cr was 0.88 with 0.79 familiarity and 0.96 criterion. Both indicated reliable results. The overall coordination coefficients of the first and second were 0.164 (*p* < 0.001) and 0.180 (*p* < 0.001), respectively, indicated that expert opinion has good coordination and the result is reliable [57].

As shown in Table 5, the 32 competences average importance score ranged from 4.20 ± 0.77 (innovative spirit) to 5.00 ± 0.00 (spirit of teamwork and symptom management ability), CV ranged from 0.00 (spirit of teamwork and symptom management ability) to 0.21 (objectivity and fairness). All competences meet retention criteria. The experts’ comments were divided into four categories: 1) strengthen the expected characteristics in competence expression and definition. For example, “spirit of teamwork” was suggested to revise the expression to strengthen the leading role of the nurse in a HPC team. 2) merge the competences that have overlapping definitions and revise the expression. For example, the definition of “critical thinking” covers that of “evaluation and analysis ability”, therefore these two competences were merged and only presented “critical thinking” in the next round. 3) revise the competence domain expression for better readability or better definition matching. In this category, the experts agreed with the inclusion of competence but suggested revising the expression by adding or deleting some words or using a synonym. For example, “evidence-based practice ability” was suggested to remove “ability” due to verbose expression. 4) no suggestions or comments. Five competences including innovative spirit, self-awareness, achievement motivation, psychological regulation, ethics and legal awareness and corresponding definitions received no revision comments. One competence, plan execution ability, was deleted due to the lack of HPC specialty. In summary, after the first round Delphi survey, 32 competence domains were reduced to 24.

**Table 5:** The importance score and comments of 32 HPC competences in the 1st round Delphi survey

Numbers	Competences	Mean	SD	CV	Comments summary	Revised competences
1	Spirit of teamwork	5.00	0.00	0.00	The nurse may not be the actual leader of the HPC team, but the nurse still needs to have a holistic view when they are working as a team member. Therefore, the competency expression may consider revised to “holistic view of teamwork”, and the meaning of “having a holistic view and giving full play to the advantages of the team” may add to the interpretation	Holistic view of teamwork (1)
2	Critical thinking	4.80	0.41	0.09	The competency “critical thinking” covers “evaluation and analysis ability”, and may consider merging these two competences and their definitions	Critical thinking (2)
3	Evaluation and analysis ability	4.87	0.35	0.07		
4	Communication ability	4.87	0.35	0.07	These two competences have overlapped parts, may consider merging them and their definitions	Interpersonal communication (2)
5	Interpersonal relationship	4.73	0.46	0.10		
6	Innovative spirit	4.20	0.77	0.18	No revision was suggested	Innovative spirit (4)
7	Evidence-based practice ability	4.60	0.63	0.14	The expression is verbose, may consider removing “ability”	Evidence-based practice (3)
8	Study ability	4.67	0.49	0.10	This competency should strengthen self-motivation, may consider adding this aspect in expression	Self-motivated learning (1)
9	Objectivity and fairness	4.27	0.88	0.21	These two competences “Objectivity and fairness” and “respect for others” are overlapped, and they are not compulsory for HPC. Therefore, may consider merging these two competences and revising to “dignity protection” and revise the definition accordingly	Dignity protection (2)
10	Respect for others	4.93	0.26	0.05		
11	Proactiveness	4.73	0.59	0.13	“Proactiveness” does not match the given definition, may consider revising it to “responsibility”	Responsibility (3)
12	Empathy	4.87	0.35	0.07	The definition of “empathy” covers “sensitivity”, may consider merging them and their definitions	Empathy (2)
13	Sensitivity	4.73	0.59	0.13		
14	Facilitate decision-making ability	4.67	0.49	0.10	The “supportive ability” included information support for decision making which can merge with the “Facilitate decision-making ability”, and mental/psychological support which can merge with “spiritual care ability”. After merging the competences, may delete “ability” to avoid verbosely	Facilitate decision-making (2)
15	Supportive ability	4.60	0.63	0.14		
16	Spiritual care ability	4.60	0.63	0.14		Mental and spiritual care (2)
17	Life education ability	4.73	0.46	0.10	The item “Life-and-death value” is not competency, may consider merging with the competency “life education ability” and deleting “ability” to avoid verbosely	Life education (2)
18	Life-and-death value	4.60	0.63	0.14		
19	Promote others’ development	4.33	0.72	0.17	“Promote others’ development” is one aspect of leadership, may consider expanding this competency concept into leadership and revise the definition accordingly	Leadership (1)
20	Self-awareness	4.47	0.74	0.17	No revision was suggested	Self-awareness (4)
21	Organization and coordination ability	4.67	0.49	0.10	The expression is verbose, may consider removing “ability”	Organization and coordination (3)
22	Achievement motivation	4.53	0.64	0.14	No revision was suggested	Achievement motivation (4)
23	Spirit of contribution	4.73	0.46	0.10	“Spirit of contribution” does not match the given definition, may consider revise to “spirit of service”	Spirit of service (3)
24	Psychological regulation	4.87	0.35	0.07	No revision was suggested	Psychological regulation (4)
25	Stress coping ability	4.87	0.35	0.07	The expression may consider revised to “stress management”	Stress management (3)
26	Symptom management ability	5.00	0.00	0.00	The definition of “pain management ability” covers “symptom management”, and may consider merging them and their definitions	Symptom management (2)
27	Pain management ability	4.93	0.26	0.05		
28	Plan execution ability	4.47	0.52	0.12	Belongs to the basic nurse ability, Deleted	/
29	Bereavement care ability	4.47	0.74	0.17	The expression is verbose, may consider removing “ability”	Bereavement care (3)
30	Basic caring ability	4.73	0.59	0.13	The “basic caring ability” does not show the HPC specialty may consider revising to “comfort care”	Comfort care (3)
31	Kindness	4.73	0.46	0.10	The competency may consider adding “friendly” and corresponding content in the definition	Kindness and friendly (3)
32	Ethics and legal awareness	4.73	0.59	0.13	No revision was suggested	Ethics and legal awareness (4)

(1) to (4) refer to the comments of strengthening the expected characteristics in competency expression and definition, merging the competences that have overlapping definitions and revising the expression, revising the competency domain expression for better readability or better definition matching, and no suggestions or comments, respectively

As shown in Table 6, in the second round Delphi survey, among the revised 24 competence domains, the average importance score ranged from 4.31 ± 0.75 (achievement motivation) to 5.00 ± 0.00 (holistic view of teamwork, symptom management, and comfort care), CV ranged from 0.00 (holistic view of teamwork, symptom management, and comfort care) to 0.17 (achievement motivation). Compared with the first round, the average importance score increased and the disagreement between experts decreased. In this round, only five competences received revision comments that were mainly about expression revision. After two rounds of the Delphi survey, 25 competences were determined. The competence “mental and spiritual care” was separated to “psychological care” and “spirit care”.

**Table 6:** The importance score and comments of 24 HPC competences in the 2nd round Delphi survey

Numbers	Competences	Mean	SD	CV	Comments summary	Revised competences
1	Holistic view of teamwork	5.00	0.00	0.00	The competency element should be more about practical actions rather than only awareness. It is recommended to strengthen the cooperation within and between teams	Interprofessional collaboration
2	Critical thinking	4.77	0.44	0.09	No revision was suggested	Critical thinking
3	Interpersonal communication	4.92	0.28	0.06	No revision was suggested	Interpersonal communication
4	Innovative spirit	4.46	0.66	0.15	No revision was suggested	Innovative spirit
5	Evidence-based practice	4.67	0.49	0.11	No revision was suggested	Evidence-based practice
6	Self-motivated learning	4.69	0.48	0.10	No revision was suggested	Self-motivated learning
7	Dignity protection	4.92	0.28	0.06	No revision was suggested	Dignity protection
8	Responsibility	4.77	0.44	0.09	No revision was suggested	Responsibility
9	Empathy	4.85	0.38	0.08	No revision was suggested	Empathy
10	Facilitate decision-making	4.69	0.48	0.10	No revision was suggested	Facilitate decision-making
11	Life education	4.85	0.38	0.08	No revision was suggested	Life education
12	Leadership	4.46	0.66	0.15	No revision was suggested	Leadership
13	Self-awareness	4.62	0.65	0.14	No revision was suggested	Self-awareness
14	Organization and coordination	4.69	0.48	0.10	No revision was suggested	Organization and coordination
15	Achievement motivation	4.31	0.75	0.17	No revision was suggested	Achievement motivation
16	Spirit of service	4.75	0.45	0.10	The contribution may be more appropriate in describing the nature of the HPC nurse's job	Spirit of contribution
17	Psychological regulation	4.85	0.38	0.08	Must identify the person of regulation	Self-psychological regulation
18	Stress management	4.85	0.38	0.08	No revision was suggested	Stress management
19	Symptom management	5.00	0.00	0.00	No revision was suggested	Symptom management
20	Bereavement care	4.69	0.63	0.13	No revision was suggested	Bereavement care
21	Comfort care	5.00	0.00	0.00	No revision was suggested	Comfort care
22	Mental and spiritual care	4.69	0.63	0.13	Mental/psychological care and spiritual care are different and should consider divided into two competences	Psychological care, spirit care
23	Kindness and friendly	4.83	0.39	0.08	The expression should be more professional and general	Affinity
24	Ethics and legal awareness	4.69	0.63	0.13	No revision was suggested	Ethics and legal awareness

The number 22 competence “mental and spiritual care” was separated to “psychological care” and “spirit care”

### The structure exploration

In the cross-sectional study, total 783 questionnaires were received, and 292 were excluded due to not signing e-informed-consent (*n* = 3), completing time < 150 s (*n* = 91), neither taking care of end-of-life patients in the past 12-month nor receiving the HPC training (*n* = 52), and giving different answers in the repeated two questions (*n* = 146), leaving 491 valid questionnaires. The participants were mostly female (*n* = 476, 96.94%), married (*n* = 352, 71.69%), having a bachelor’s degree (*n* = 378, 76.99%), and working in general or cancer hospitals (*n* = 398, 81.06%). One fifth of the participants (*n* = 95, 19.35%) were qualified HPC specialist nurse, others were registered nurse (RN) whose work involves HPC. Most of the participants were working in clinic (*n* = 369, 75.15%) while 82 (16.70%) were engaged in management being nurse manager or the director of nursing department. The self-report working time ranged from 6 months to 40 years.

The average importance scores and CV of all 25 competence domains were > 3.00 (ranged from 4.71 to 4.87) and < 0.25 (ranged from 0.08 to 0.12), respectively (Table 7), indicating the reaching of consensus. The Kaiser–Meyer–Olkin measure verified the sampling adequacy for the exploratory factor analysis, KMO = 0.973. Bartlett’s test of Sphericity χ^2^(300) = 17277.958, *p* < 0.001, indicating that correlation structure is adequate for factor analyses. The maximum likelihood factor analysis with a cut-off point of 0.40 and the Kaiser’s criterion of eigenvalues > 1 [59, 60] yielded a two-factor solution as the best fit for the data, accounting for 79.30% of the variance. The results were presented in Table 7. The factor 1 consisted of 18 competences contributing to direct care and self-regulation, therefore named as Basic Competences. The factor 2 consisted of 7 competences contributing to advanced practice and career development named as Developmental Competences.

**Table 7:** The importance score and theoretical underline structures of HPC competences framework for Chinese nurse

Competency domains	Mean	SD	CV	Rotated matrix of two factors		Factors
				**1**	**2**	
Responsibility	4.87	0.38	0.08	**0.87**	0.35	Factor 1: Basic Competences
Self-psychological regulation	4.87	0.38	0.08	**0.87**	0.37	
Interpersonal communication	4.86	0.39	0.08	**0.84**	0.41	
Stress management	4.85	0.39	0.08	**0.82**	0.38	
Comfort care	4.86	0.40	0.08	**0.81**	0.48	
Dignity protection	4.85	0.40	0.08	**0.80**	0.44	
Psychological care	4.85	0.42	0.09	**0.79**	0.50	
Self-awareness	4.82	0.42	0.09	**0.79**	0.44	
Empathy	4.82	0.43	0.09	**0.78**	0.37	
Affinity	4.83	0.42	0.09	**0.76**	0.44	
Life education	4.84	0.42	0.09	**0.74**	0.48	
Spirit of contribution	4.81	0.45	0.09	**0.74**	0.39	
Symptom management	4.83	0.45	0.09	**0.74**	0.40	
Spirit care	4.84	0.43	0.09	**0.74**	0.54	
Interprofessional collaboration	4.79	0.47	0.10	**0.70**	0.51	
Self-motivated learning	4.80	0.46	0.10	**0.67**	0.59	
Bereavement care	4.77	0.51	0.11	**0.58**	0.55	
Facilitate decision-making	4.75	0.52	0.11	0.40	**0.83**	Factor 2: Developmental Competences
Achievement motivation	4.71	0.57	0.12	0.31	**0.83**	
Leadership	4.71	0.56	0.12	0.38	**0.82**	
Innovative spirit	4.74	0.54	0.11	0.39	**0.82**	
Organization and coordination	4.75	0.52	0.11	0.45	**0.77**	
Evidence-based practice	4.78	0.48	0.10	0.52	**0.69**	
Ethics and legal awareness	4.78	0.51	0.11	0.46	**0.67**	
Critical thinking	4.78	0.47	0.10	0.60	**0.65**	

Extraction method was principal component analysis; rotation method was varimax with Kaiser normalization; loadings larger than 0.40 are in bold

## Discussion

This mixed-method study presented the process of constructing a HPC competence framework for nurses and revealed it had a two-factor structure. We observed in systematic review that although hospice care has evolved for several decades, the HPC related competence had few changes. The earliest paper [17] recruited in systematic review was published in 1995 by American scholars suggesting 8 HPC nurse competences, including connecting competence, encouraging choice, speaking truth, strengthening the family, comforting, spiritual caring, guiding letting go [17]. Excepting “speaking truth” that has become a legal requirement and has been rarely mentioned in the subsequent studies, other 7 competences suggested by [17] were continually listed in different expressions as general or HPC nurses’ competences in later research articles, especially Chinese articles (Additional file 3). For example, the connecting competence was later presented as communication competence/abilities, [36] or as multi-professional collaboration competence/ ability [15]. This fact may prevent us to estimate optimistically the development of HPC nurses’ competence in the recent two decades. Meanwhile, it further revealed the necessity of constructing a standardized HPC competence framework, that may help reduce the duplication of synthesis efforts by scholars in different nations or regions as well as provide clear competence goals for human resource training in the HPC field.

The competence profile “adaptive ability” and “psychological regulation ability” extracted from the interview were not reported in the articles recruited in the systematic review. In addition, the “psychological regulation” also retained in the Delphi survey. These results indicated that Chinese HPC nurse and experts agreed the importance this competence. From the 13 interviewees, we gathered that they were simultaneously providing care to both end-of-life patients and regular patients, irrespective of their working department. The nurses had to constantly switch the role between encouraging the patients pursuing active treatment and helping dying patients pursing inner peace. Here was a quote from one of the interviewees to show the psychological struggles.

*“…… It is very different, although the communication skills are same, the direction of is different. If the patient is receiving anti-tumors treatment, we have to maintain his/her willingness and confidence on treatment. The patient may often say “I can't be treated”, and then the doctors and nurses will naturally say “you are fine, you will try and then you will be better”. But, for those receiving HPC care, you may have to help him/her thinking about what he/she has to do or to prepare when he/she is dying. The direction is different. So, this is why that many nurses in hospice, especially all the nurses who are new to hospice, will be upset and frustrated most of the time ……”*

The role conflicts may increase the working pressure and the psychological burden of nurses [61]. Considering this situation may not change in a short time, the HPC competence framework constructed in this study can be a suitable foundation of Chinese HPC nurse selection and training. Additionally, our research confirmed the importance of including the views of frontline staff when constructing a competence framework.

As a result of two-round Delphi survey, among all competences, the “achievement motivation” was assessed at the lowest average importance score (4.31 ± 0.75) and the largest CV (0.17). Some experts regarded this competence belonged to a relative higher level that the HPC nurses may not really use it in daily work. Other experts and our research team decided this competence should be retained considering the future implementation of the constructed framework. McClelland published an article in 1973 discussed that one of the central pieces of evidence about competence replacing intelligence was the impact of achievement, [62] showing that achievement motivation was a very implicit but very powerful intrinsic contributor to competency. This competence framework was developed for different level of HPC nurses, including RN, APN, and nurse manager, some level may need this competence. Meanwhile, this framework may be used for assessment tool development, having domain like “achievement motivation” may help distinguish individuals competence evaluation.

The competences that were repeatedly modified in the two rounds of Delphi surveys were “interprofessional collaboration”, “spirit of contribution”, “psychological care” “spirit care”, “self-psychological regulation”, and “affinity”. The “interprofessional collaboration” was revised from “spirit of teamwork”. As having the spirit of teamwork is the foundation of interprofessional collaboration, this revision increased the requirements of nurses. Although in China the nurses may not be the actual leaders of HPC teams, in real world the nurses are the absolute core working force and the persons who know the patients and caregivers the best [63]. Therefore, the nurse should have beyond team-member-level competence. The modification of the other five competences is mainly the improvement of expression, and will not be discussed further here.

The exploratory factor analysis showed our HPC competence framework had two factors. Based on the authors’ observation of clinic work the 18 competences in the Basic Competences factor directly contributed to the nurses’ daily work while the 7 competences in the Developmental Competences factor contributed more to high requirement aspects such as, research and self-continuous development. The importance scores of factor Basic Competences were higher than that of Developmental Competences may indicated that currently the Chinese clinical nurses focused more in completing daily work with high quality. The importance score of all competences ≥ 4.71 (out of 5) indicated Chinese clinical nurses have realized both Basic Competences and the Developmental Competences were important. According to these results, when using our HPC competence framework to guide the training program design, the organizers could consider based on the qualifications of trainee selecting the competences in on factor as the main training content. Or the organizers could make the training series, from the improving the Basic Competences to more advanced Developmental Competences. The overseas HPC frameworks supported the above suggestions because three frameworks recruited in the systematic review categorized competences. The America and New Zealand HPC framework [23, 24] categorized competences to the core competences that all nurses should master and specialty competences that only nurses working in palliative specialty environment were needed. The United Kingdom HPC framework [43] gave fixed seven competency areas, but with progressive competency requirements for increasing qualification level of nurses. Although the Australia [44] and Canada framework [20] listed several competency domains and give unified requirements, these two frameworks were designed only for HPC specialized nurses. In summary, although the specific content of the frameworks in different countries was different, structuring the competences was a unified trend.

**Fig. 3:**
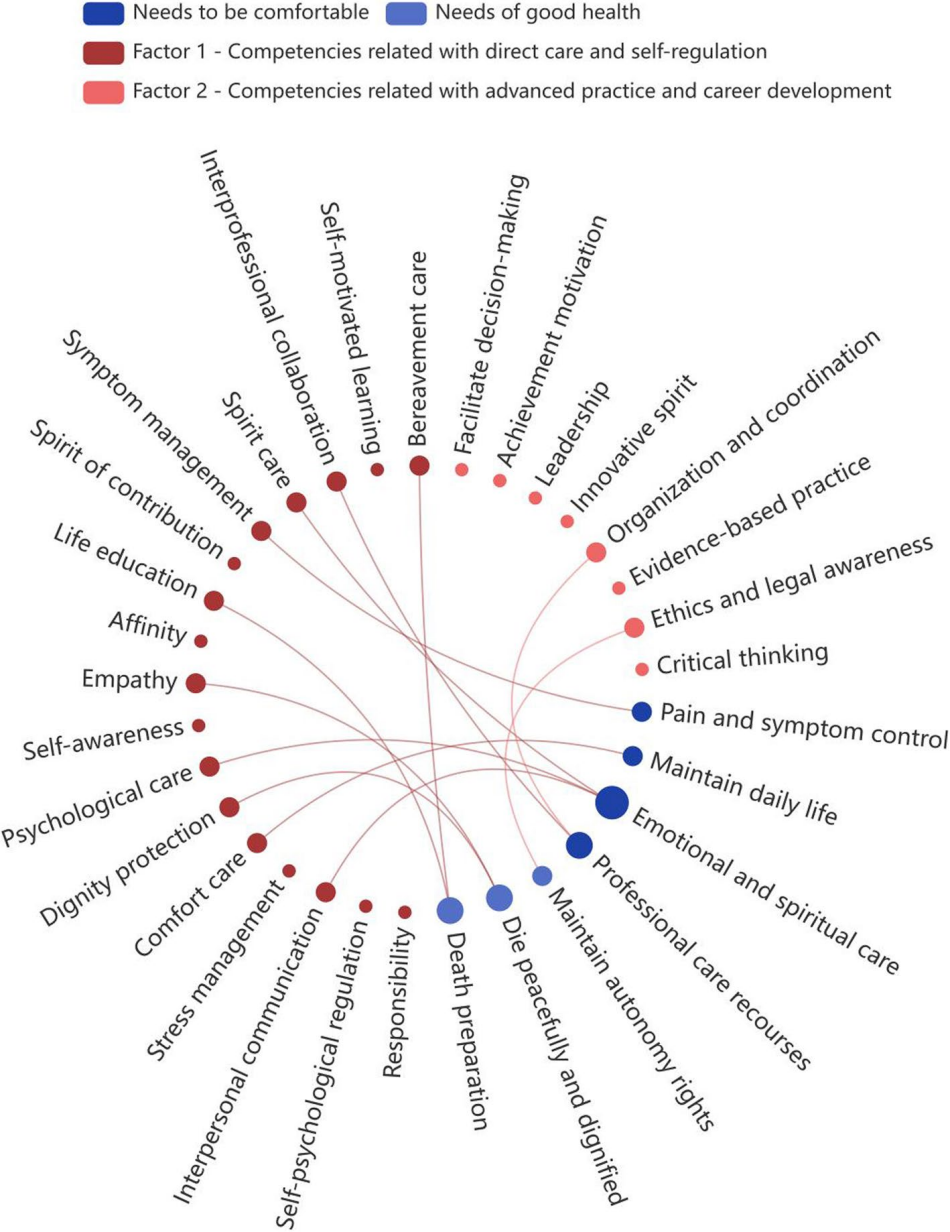
The constructed HPC competence framework for nurse covers the needs of Chinese dying patients and their family caregivers

Implementation value of our HPC competence framework also proved by covering the HPC needs of Chinese patients. A previous systematic review summarized seven care needs of Chinese dying patients and their family caregivers (Fig. 3) [64]. The competences for meeting these needs are all covered by our competence framework. For example, the patients have the need of “pain and symptom control” that is matched by “Symptom management” and “Comfort care” in the current framework. Other possible match relationships are shown in Fig. 3. Therefore, it can be foreseen that the nurses trained under this HPC competence framework will be capable providing proper care of Chinese dying patients and their family caregivers. In addition, nurses working in HPC field may use the framework to do self-assessment, identify future training needs, and even guiding the career development. Hospitals or health-care facilities may use the framework as guide to develop an appraisal system assessing staff’s competence or being the basis of the human resource allocation.

From a design perspective, our study extracted competencies from international publications and synthesized the attitudes of two key stakeholders: academic and clinical experts in the HPC field, and nurses working on the HPC frontline. Therefore, we are confident in stating that this 25-competencies framework has broad applicability, and nurses trained with these competencies as a target should be competent in clinical palliative care work in most countries. Furthermore, we have grouped the 25 competencies into Basic Competences and Developmental Competences based on clinical nurses' ratings. This grouping suggests that a continuous HPC education program may consider a two-stage design. We propose including Basic Competences in HPC training for general nurses or in the initial training for novice HPC nurses. Developmental Competences may be reserved for the continuous education program targeting experienced HPC nurses seeking career development.

The strength of this study included 1) extracting competence from multi-resources including published literatures, clinical nurses’ interviews, and experts’ opinions, maximized the comprehensiveness and clinical usefulness of the extracted content; 2) exploring the underline structure of the constructed HPC competence framework that deepened the understanding of the Chinese nurses’ attitude on HPC competences, and increase the implementation potential of the framework. This study has a limitation due to the nature of Delphi survey. Although the Delphi panel members had diverse background and rich experience in HPC, they may not be adequate to represent all opinions of the HPC experts in China. Therefore, the online survey recruiting a large sample was conducted for more opinion collection. Another limitation was that clinical nurses may not have a holistic view or a clear understanding of the future HPC development in China, so the structure of the competency framework derived from the clinical nurse perspective alone may need to be modified in consultation with relevant government department officers and experts in the HPC field. Considering that clinical nurses reflected current realities of clinical palliative and hospice care, it was recommended using the known framework structure until further results are available.

## Conclusion

By extracting and synthesizing HPC related competence profiles for nurses from publications and interview transcripts and pursuing consensus from experts, we constructed a 25 HPC competence framework for nurses. This framework may have two underline factors that one about basic competence (18 competences) contributing to direct care and self-regulation, another one about developmental competence (7 competences) contributing to advanced practice and career development. This framework has promising implementation potential that researchers may consider using it as foundation to develop competence assessment or self-evaluation tools and education programs.

### Supplementary Information

**Additional file 1.** Systematic review searching keywords and strategy.

**Additional file 2.** Qualitative Interview Outline.

**Additional file 3.** Table of evidence of the systematic review.

## Data Availability

The original data is encrypted and stored according to the requirements of the corresponding author's university. We intend to share data (with private information removed) for non-profit purposes with researchers interested in this study and obtained the permission of corresponding author's university.

## Abbreviations

HPC: Hospice and Palliative Care
EFA: Exploratory Factor Analysis
Cr: Authority Coefficient
