# Supplementary material for: Building bridges of excellence: a comprehensive competence framework for nurses in hospice and palliative care—a mixed method study
Source: BMC Palliat Care. 2023 Dec 12;22:197. doi: 10.1186/s12904-023-01318-x (PMC10714629; doi:10.1186/s12904-023-01318-x)
Supplement: Supplementary file 1 — Additional file 1. Systematic review searching keywords and strategy. [file 12904_2023_1318_MOESM1_ESM.docx]

**Additional file 1 Systematic review searching keywords and strategy**

The keywords, searching strategy, selection criteria used in two rounds systematic review were same.

- **Key words**

Table 1 Searching key words

| **Theme** | **English** | **Chinese** |
| --- | --- | --- |
| Hospice and palliative care theme | palliative care OR hospice care OR end-of-life care OR terminal care | 安宁疗护 OR临终关怀OR 终末期护理OR 临终护理OR 姑息治疗 OR 姑息护理 |
| Competency theme | competenc* OR professional competenc* OR skills OR abilit* | 胜任力 OR 能力 OR 技能 |
| Target population theme | nurs* OR advanced practice nurs* | 护士 |

- **Database & searching strategy**

Six databases, PubMed, CINAHL Plus, PsycINFO, WanFang Data (Chinese), and CNKI (Chinese), and SinoMed (Chinese) were searched for peer-reviewed articles, national/ official documents, and gray literature reports using the following query: *(palliative care OR hospice care OR end-of-life care OR terminal care) AND (competenc* OR professional competenc* OR skills OR abilit*) AND (nurs* OR advanced practice nurs*)*. The publication time were set as between January 1990 to February 2020 and February 2020 to February 2022, respectively. The languages limited to Chinese and English. All related Medical Subject Headings (MeSH) were included. Hand search on the reference lists of all recruited papers were also performed.

- **Inclusion/exclusion criteria**

The inclusion criteria were: 1) focusing population including registered nurse or advanced practice nurse or nurse manager; 2) exploring or discussing palliative care related competences or abilities; 3) being peer-reviewed research papers, national or official documents/reports, and diploma thesis; 4) full-text available; 5) writing in English or Chinese. The exclusion criteria were: 1) focusing on nursing students, groups/organizations, or health care professionals other than nurse; 2) grey literature other than diploma thesis.

- **Data extraction and critical appraisal**

Two authors (Liang & Fang) independently extracted author, year, country, article type/ study design, target nurse, and ability/ competence profiles and corresponding context (if any) from 30 recruited publications. Disagreements were judged by another author (Zhu). The extracted competence profiles were integrated by authors referring to the known competence dictionaries. All authors have researched a consensus on the integrated 87 competence profiles (Table 2). Because the recruited the articles were under qualitative synthesis and we recruited master or doctor thesis, we implemented objective quality evaluation by considering the journal level, author/associations’ background, and publications’ content rather than followed existed critical appraisal tool.

- **Registration**

We did not register the study protocol.
