# Supplementary material for: Building bridges of excellence: a comprehensive competence framework for nurses in hospice and palliative care—a mixed method study
Source: BMC Palliat Care. 2023 Dec 12;22:197. doi: 10.1186/s12904-023-01318-x (PMC10714629; doi:10.1186/s12904-023-01318-x)
Supplement: Supplementary file 2 — Additional file 2. Qualitative Interview Outline. [file 12904_2023_1318_MOESM2_ESM.docx]

**Additional file 2 Qualitative Interview Outline**

Dear Nursing Colleagues：

We are conducting a study on investigating the hospice and palliative care nurse competence and hope to identify the competence profiles from key events. The two main questions in this interview were: 1. What are the two most successful things you have done at work and 2. What are the two most unsuccessful things you have done at work? Please follow the outline below and describe the events in as much detail as possible. Thank you for your cooperation!

Note: Please describe what actually happened, as requested, not hypothetical situations or just ideas you agree with, behaviors you approve of, etc. You may wish to think back for a few minutes before describing, or write down what you feel is important, as this will help you to describe better.

**Part I: The two things you feel most successful in your work**

As a hospice nurse, there are many things that you do daily that you consider to be very successful and are proud of. You can follow the outline below to describe them in detail.

1. When and where did this event take place? (Approximate date and location)

2. What was the reason, history and ending of the incident? (The more details the better)

3. Who was involved in it? (You can replace the specific name with the job title)

4. What were your feelings or thoughts at the time? (You can describe your personal emotions such as panic, confidence, your view of the incident, your view of the people involved, etc., both positive and negative)

5. What did you do or say at the time and what actions did you take? How did you know what to do or say?

6. What did you learn from this incident?

7. If a similar situation occurs again, how will you handle it? Why?

**Part 2: The two things you feel are your biggest failures at work**

Hospice work may also bring you frustration. As a nurse working in hospice nursing, what are some of the things that you feel are failures in your daily work that could be some inspiration and warning to those working in related nursing? You can follow the outline below to elaborate.

1. When and where did this event take place? (Approximate date and location)

2. What was the reason, history and ending of the incident? (The more details the better)

3. Who was involved in it? (You can replace the specific name with the job title)

4. What did you feel or think at the time? (You can describe your personal emotions such as panic, confidence, your view of the incident, your view of the people involved, etc., both positive and negative)

5. What did you do or say and what actions did you take? How did you know what to do or say?

6. What did you learn from this incident?

7. If a similar situation occurs again, how will you handle it? Why?
