# Supplementary material for: Building bridges of excellence: a comprehensive competence framework for nurses in hospice and palliative care—a mixed method study
Source: BMC Palliat Care. 2023 Dec 12;22:197. doi: 10.1186/s12904-023-01318-x (PMC10714629; doi:10.1186/s12904-023-01318-x)
Supplement: Supplementary file 3 — Additional file 3. Table of evidence of the systematic review. [file 12904_2023_1318_MOESM3_ESM.docx]

| **Additional file 3 Table of evidence of the systematic review** | | | | |
| --- | --- | --- | --- | --- |
| **Author** | **Study design**  **Article type** | **Target nurse** | **Competence profiles/domains** | **Manuscript context** |
| Joyce,  1995, America | qualitative study | Hospice nurses | Connecting competency | being there |
|  |  |  |  | hearing and asking |
|  |  |  |  | deliberately building trust |
|  |  |  | Encouraging choice | not specified in the article |
|  |  |  | Speaking truth | not specified in the article |
|  |  |  | Collaborating | not specified in the article |
|  |  |  | Strengthening the family | assessing |
|  |  |  |  | developing ability |
|  |  |  |  | going between |
|  |  |  |  | facing limitations |
|  |  |  | Comforting | providing hands on care |
|  |  |  |  | anticipating comfort needs |
|  |  |  |  | trying multiple options |
|  |  |  |  | balancing pharmacological effects |
|  |  |  |  | organizing and reorganizing regimens |
|  |  |  |  | making major changes in regimens |
|  |  |  |  | initiating nontraditional therapies |
|  |  |  |  | facing limits |
|  |  |  | Spiritual caring | recognizing spiritual issues |
|  |  |  |  | dialogue about spiritual issues |
|  |  |  |  | fostering reconciliation |
|  |  |  |  | sharing nearing death experiences |
|  |  |  | Guiding letting go | letting go of former hopes and activities |
|  |  |  |  | letting go of life itself |
|  |  |  |  | predicting imminent death |
|  |  |  |  | being present at death |
| Becker, 2000, America | literature review; objective critique for experts;  be piloted in specialist unit | Nurses in palliative care | Communication skills | not specified in the article |
|  |  |  | Psychosocial skills | not specified in the article |
|  |  |  | Teamwork skills | not specified in the article |
|  |  |  | Physical care skills | not specified in the article |
|  |  |  | Life closure skills | not specified in the article |
|  |  |  | Intrapersonal skills | not specified in the article |
| Wright, 2001, America | qualitative study | Hospice nurses | Enough knowledge | end-stage disease process |
|  |  |  |  | signs of impending death |
|  |  |  |  | palliative therapeutics |
|  |  |  |  | collaboration between disciplines |
|  |  |  |  | advocacy |
|  |  |  |  | philosophy and ethics of hospice care |
|  |  |  |  | family dynamics |
|  |  |  | Assessment skills | not specified in the article |
|  |  |  | Communication skills | not specified in the article |
|  |  |  | Technical skills | not specified in the article |
|  |  |  | Management skills | not specified in the article |
| Desbiens, 2011, Canada | literature review; individual interviews; focus group  discussion | Nurses who care for adult patients and their family experiencing a  life-limiting illness or  at end of life across various healthcare  settings or in the community. | Pain assessment and management | assess pain in a Palliative care patient considering all of the following elements: quality, duration, location, intensity, exacerbating and alleviating factors |
|  |  |  |  | assess pain for a Palliative care patient unable to communicate |
|  |  |  |  | effectively use pharmacological interventions to alleviate pain in a Palliative care patient |
|  |  |  |  | use nonpharmacological and complementary interventions to alleviate pain in a Palliative care patient |
|  |  |  |  | provide early detection of side effects related to pain medication. |
|  |  |  | Other symptoms management | provide effective care to alleviate nausea and vomiting in a Palliative care patient |
|  |  |  |  | provide effective care to alleviate constipation in a Palliative care patient |
|  |  |  |  | provide effective care to alleviate fatigue in a Palliative care patient |
|  |  |  |  | provide effective care to alleviate dyspnea in a Palliative care patient |
|  |  |  |  | provide proper mouth care to promote comfort in a Palliative care patient |
|  |  |  | Psychological care | provide early detection of delirium in a Palliative care patient |
|  |  |  |  | assess depression in a Palliative care patient and their family |
|  |  |  |  | provide effective care to reduce psychological distress in a Palliative care patient and their family |
|  |  |  |  | assist a Palliative care patient and their family to cope with stressors related to the illness |
|  |  |  |  | provide support to a Palliative care patient and their family when they experience grief. |
|  |  |  | Social support | assess the impact of a life-limiting illness on family dynamics |
|  |  |  |  | assist a Palliative care patient and their family in maintaining cultural traditions despite illness |
|  |  |  |  | assist a Palliative care patient and their family in identifying personal resources to cope with problems related to a life-limiting illness. |
|  |  |  |  | promote communication between a Palliative care patient and their family members when a conflict occurs |
|  |  |  |  | refer a Palliative care patient and their family to appropriate resources to meet their social needs |
|  |  |  | Spiritual care | assess the spiritual needs of a Palliative care patient and their family |
|  |  |  |  | recognize signs of spiritual distress in a Palliative care patient and their family. |
|  |  |  |  | help a Palliative care patient and their family to explore various sources of hope when they demonstrate signs of hopelessness |
|  |  |  |  | assist a Palliative care patient to explore the meaning of their illness experience |
|  |  |  |  | adapt the nursing care in accordance with the spiritual beliefs of a Palliative care patient and their family |
|  |  |  | Meet the needs related to functional status of patients and caregivers | assess the needs associated with activities of daily living in a Palliative care patient |
|  |  |  |  | assess the need for functional support to prevent burnout in family members caring for a Palliative care patient |
|  |  |  |  | assist a Palliative care patient to maintain their functional independence for as long as possible |
|  |  |  |  | empower family members to provide care to a Palliative care patient |
|  |  |  |  | implement appropriate interventions to help alleviate burden on family members caring for a Palliative care patient |
|  |  |  | Ethical and legal issues | promptly identify ethical issues related to the care of a Palliative care patient |
|  |  |  |  | provide information to a Palliative care patient concerning the legal issues associated with life-limiting illness |
|  |  |  |  | assist a Palliative care patient to make informed decisions regarding end-of-life care |
|  |  |  |  | advocate for a Palliative care patient and their family with other members of the healthcare team |
|  |  |  |  | advocate for a Palliative care patient when there is a difference in perspective with their family on a PC issue |
|  |  |  | Interprofessional collaboration and communication | actively participate in discussions regarding a PC clinical situation during interdisciplinary team meetings |
|  |  |  |  | promote communication between healthcare professionals regarding a Palliative care patient to support continuity of care |
|  |  |  |  | promote communication between the Palliative care patient, their family, and healthcare professionals to ensure information sharing |
|  |  |  |  | promote communication between healthcare professionals when conflicts arise in the care of a Palliative care patient and their family |
|  |  |  |  | promote the collaboration of various healthcare professionals in the care of a Palliative care patient and their family |
|  |  |  | Personal and professional issues related to nursing care | recognize how my own personal and professional beliefs may influence the care I provide to a Palliative care patient and their family |
|  |  |  |  | cope with loss and grief related to the care of a Palliative care patient and their family |
|  |  |  |  | identify which stressors affect me when I provide care to a Palliative care patient and their family |
|  |  |  |  | identify your own personal resources to manage stress related to caring for Palliative care patient and their family |
|  |  |  |  | discuss death and dying with a Palliative care patient and their family |
|  |  |  | End-of-life care | provide effective care to relieve pain during the last hours of life. |
|  |  |  |  | provide effective care to relieve respiratory distress during the last hours of life |
|  |  |  |  | identify the signs and symptoms of imminent death |
|  |  |  |  | provide an authentic presence during the last hours of life to a Palliative care patient and their family |
|  |  |  |  | encourage expression of cultural and religious traditions for a Palliative care patient and their family during the last hours of life |
| Jacono, 2011,  Canada | literature review；web-based survey；  a national  symposium  experts survey | Nurses with undergraduate diploma | Exhibit skill in conducting holistic individual and family assessments | pain management |
|  |  |  |  | symptom management |
|  |  |  | Demonstrate knowledge and skill in managing pain and symptoms | not specified in the article |
|  |  |  | Possess requisite communication skills and an ability to engage in end-of-life decision making and planning and artfully and gracefully negotiate modes of care on an ongoing basis. | not specified in the article |
|  |  |  | Possess knowledge of cultural and spiritual issues and the ability to recognize and attend to meaning in suffering. | not specified in the article |
|  |  |  | Demonstrate ability to assess and attend to individual/family psychosocial and practical issues such as discharge planning. | not specified in the article |
|  |  |  | Shows evidence of an ability to collaborate effectively within an integrated inter-professional team. | not specified in the article |
|  |  |  | Possess knowledge and skills in recognizing and attending to ethical issues. | not specified in the article |
|  |  |  | Recognize and respond to the unique needs of special populations, eg. elders, children, those with cognitive impairments, unique and marginalized populations. | not specified in the article |
|  |  |  | Demonstrate caring for self while supporting others in their grief and bereavement. Real compassion is uplifting and contributes to personal growth: unresolved grief causes pain which can contribute to fatigue. The nurse recognizes his or her limitations and issues that could contribute to burnout. | not specified in the article |
|  |  |  | Possess awareness of the full range and continuum of palliative/end-of-life services and the settings in which they are available. | not specified in the article |
|  |  |  | Educate and train patient and family on care needs, identify the need for respite for family members, and safely and appropriately delegate care to other caregivers. | not specified in the article |
| White, 2012, America | questionnaire  survey | Hospice and palliative  nurses | Symptom management | not specified in the article |
|  |  |  | Pain control techniques | not specified in the article |
|  |  |  | How to talk to patients/families about death and dying | not specified in the article |
|  |  |  | Knowledge of palliative care | not specified in the article |
|  |  |  | Knowledge of hospice | not specified in the article |
|  |  |  | Dealing with your own feelings | not specified in the article |
|  |  |  | Recognizing impending death (physiological changes) | not specified in the article |
|  |  |  | Ethical issues (pertaining to nurse’s role) | not specified in the article |
|  |  |  | Advance directives | not specified in the article |
|  |  |  | Dealing with angry dying patients/families | not specified in the article |
|  |  |  | Legal issues (pertaining to nurse’s role) | not specified in the article |
|  |  |  | Religious and cultural perspectives | not specified in the article |
| Kang,  2013,  Korea | literature review;  Delphi study;  consensus meeting | Hospice and palliative care professionals:  physicians, nurses, social  workers, spiritual care providers (only the11  domains for nurses were  extracted in this table) | The definition of palliative care and geriatrics | not specified in the article |
|  |  |  | Physical care | pain management, symptom management, psychical care |
|  |  |  | Complementary intervention | not specified in the article |
|  |  |  | Family care | not specified in the article |
|  |  |  | Spiritual care | not specified in the article |
|  |  |  | Communication | not specified in the article |
|  |  |  | Management and quality assurance | not specified in the article |
|  |  |  | Pediatrics & adolescent care | not specified in the article |
|  |  |  | Psychological symptom care | not specified in the article |
|  |  |  | Ethics in palliative care | not specified in the article |
|  |  |  | Education and research | not specified in the article |
| Tania,  2016, Germany | experts’ conclusion  based on  international  association for  hospice and  palliative care list of  essential practices;  Delphi study | Undergraduate level  nurses and physicians | Definition and principles of palliative care | definition and principles of palliative care |
|  |  |  |  | models of palliative care |
|  |  |  | Identification and control of  symptoms | pain |
|  |  |  |  | respiratory symptoms |
|  |  |  |  | gastrointestinal symptoms |
|  |  |  |  | insomnia |
|  |  |  |  | delirium |
|  |  |  | End-of-life care | palliative sedation |
|  |  |  |  | end-of-life care |
|  |  |  | Ethical and legal issues | not specified in the article |
|  |  |  | Psychosocial and spiritual issues | emotional issues |
|  |  |  |  | grief and bereavement |
|  |  |  |  | spirituality |
|  |  |  |  | caregiver and family |
|  |  |  |  | communication and therapeutic relationship |
|  |  |  | Teamwork | not specified in the article |
| Yamani, 2018,  Iran | interviews;  Delphi study;  literature review;  an expert panel | Oncology nurses | Holistic physical healthcare | recording of medical history and physical examination |
|  |  |  |  | respiratory system, GI system, cardiovascular system, and kidney and urinary tract care |
|  |  |  | Psychological and social care | care in patients with newly diagnosed cancer, caring for patients with end-stage cancer, and breaking bad news. |
|  |  |  | Spiritual care | improving the patient-God connection, and religious involvement |
|  |  |  | Palliative care | managing pain, caring for patients with end-stage phase disease |
|  |  |  | Ability to prevent cancer | preventing cancer, providing rehabilitation programs for patients, and participating in cancer screening programs |
|  |  |  | Teamwork and inter-professional competency | ability to work with other team members to improve the quality of care, and team accountability |
|  |  |  | Management and leadership competency | identifying, recording, tracking, evaluating, and prioritizing gaps in services, and the ability to lead and coordinate the activities of the team |
|  |  |  | Ability to conduct research and evidence-based nursing | finding appropriate information sources, and assessing the evidence |
|  |  |  | Supportive care | preventing, controlling, and relieving the side effects of disease and treatments, and improving the patients’ comfort and quality of life |
|  |  |  | Communication skills | active listening skills, effective body language, and empathy skills |
|  |  |  | Professionalism | allocating sufficient time to meet and consult with patients and their families, and commitment to honesty and integrity, research, education, and care ethics. |
|  |  |  | Educating and counseling patients and their families | applying the principles of patient education, and using appropriate teaching methods (lectures, group discussions, etc.) |
|  |  |  | Reasoning, problem-solving, and critical thinking skills | applying the principles of critical thinking in dealing with various claims (spoken, written, and visual), identifying problems related to their work, analyzing problems, and developing appropriate solutions |
| Chen,  2019,  China | patient service  demand survey;  Delphi study;  analytic  hierarchy  process (AHP) | Hospice care  specialized nurses | Assessment and decision-making skill | nursing assessment competency |
|  |  |  |  | nursing decision-making skill |
|  |  |  | Professional nursing competency | symptom control care capability |
|  |  |  |  | comfort care capability |
|  |  |  | Nursing management competency | ward management capability |
|  |  |  |  | business mentoring capability |
|  |  |  |  | communication skill |
|  |  |  | Human care capability | emotional support capability |
|  |  |  |  | death education capability |
|  |  |  | Professional development capability | learning capability |
|  |  |  |  | teaching and research ability |
| Li,  2019,  China | Delphi study;  analytic  hierarchy  process (AHP) | Palliative care nurse | Cognitive related competence | professional identity |
|  |  |  |  | professionalism |
|  |  |  |  | Prudence |
|  |  |  |  | research capability |
|  |  |  |  | self-directed learning capability |
|  |  |  |  | health education capacity |
|  |  |  | Emotion related competence | effective communication skills |
|  |  |  |  | multidisciplinary teamwork capability |
|  |  |  |  | human care capability |
|  |  |  |  | social volunteering capacity |
|  |  |  |  | stress management skill |
|  |  |  |  | emotion management skill |
|  |  |  |  | empathic capacity |
|  |  |  | Other competence | symptom assessment and management capability |
|  |  |  |  | pain assessment and management capability |
|  |  |  |  | end-of-life care competency |
|  |  |  |  | comfort care capability |
|  |  |  |  | dignity care capability |
|  |  |  |  | spiritual care capability |
|  |  |  |  | identifying patient risk capability |
|  |  |  |  | care outcome evaluation capability |
|  |  |  |  | nursing handover capability |
|  |  |  |  | bereavement counseling capability |
|  |  |  |  | legal ethics compliance capability |
|  |  |  |  | multicultural nursing competency |
|  |  |  |  | care management capability |
|  |  |  |  | clinical thinking skill |
|  |  |  |  | information management capability |
| Zhu,  2019,  China | literature review;  qualitative study;  Delphi study;  questionnaire survey | Oncology nurses | Hospice and palliative care  knowledge | humanistic knowledge |
|  |  |  |  | specialized knowledge |
|  |  |  | Communication skill | basic communication skill |
|  |  |  |  | difficult communication skill |
|  |  |  | Educational capability | life education |
|  |  |  |  | basic knowledge education |
|  |  |  | Supportive care | physiological support |
|  |  |  |  | psychological support |
|  |  |  |  | spiritual support |
|  |  |  |  | family and social support |
|  |  |  | Self-development | summarization and innovation |
|  |  |  |  | continuing education |
|  |  |  | Personal characteristics | attitude |
|  |  |  |  | personality quality |
|  |  |  | Interpersonal relationships | nurse-patient relationship |
|  |  |  |  | teamwork |
| Han,  2020,  China | qualitative study;  literature review;  Delphi study | Hospice nurses | Ethical care capability | treat patients and families equally |
|  |  |  |  | maintain the dignity of patients and families |
|  |  |  |  | respect the customs and religious beliefs of patients and their family members |
|  |  |  |  | respect the wishes of patients and families to be informed of their condition |
|  |  |  |  | respect the treatment wishes of patients and their families |
|  |  |  |  | respect and understand the patient's subjective feelings (e.g., pain, discomfort) |
|  |  |  |  | provide a quiet, comfortable, and private environment for patients and their families members |
|  |  |  |  | protects private content by patients and their families members |
|  |  |  | Clinical practice skills | be able to care for patients based on the results of identification and assessment |
|  |  |  |  | be able to promptly recognize negative emotions in patients of different ages |
|  |  |  |  | be able to deal with patients' psychological problems in a prompt and effective manner according to their personal characteristics |
|  |  |  |  | be able to promptly identify negative emotions in patients' family members of different ages |
|  |  |  |  | be able to deal with psychological problems of family members in a timely and effective manner according to their personal characteristics |
|  |  |  |  | help patients' families cope with pain of bereavement |
|  |  |  |  | be able to actively seek help from counselors to solve complex psychological problems of patients and their family members |
|  |  |  |  | be able to assess patients' social support status |
|  |  |  |  | be able to provide accurate and complete information to patients and their family members |
|  |  |  |  | be able to assess spiritual needs of patients |
|  |  |  |  | be able to use spiritual care methods to meet the spiritual needs of patients |
|  |  |  |  | be able to assess the spiritual needs of the patient's family |
|  |  |  |  | be able to use spiritual care methods to meet the spiritual needs of patients' families |
|  |  |  |  | be able to implement death education for patients of different ages |
|  |  |  |  | be able to implement death education for families of patients of different ages |
|  |  |  |  | be able to actively seek professional help to resolve complex spiritual issues of patients and their family members |
|  |  |  |  | be able to communicate information about hospice and palliative care to patients and their family members |
|  |  |  |  | be able to consult with patients, family members and multidisciplinary team to develop care program |
|  |  |  | Self-adjustment skills | have empathy while communicating with patients and their family members |
|  |  |  |  | be able to rationally control emotions in the face of negative psychological reactions of patients and their family members |
|  |  |  |  | be able to identify faced problems such as career havoc/energy depletion |
|  |  |  |  | be able to self-regulate the impact of negative emotions on work |
|  |  |  |  | be able to use social support resources to regulate work stress |
|  |  |  |  | be able to fairly understand and evaluate roles in work and maintain a normal work mood |
| Li,  2020,  China | literature review;  qualitative research;  Delphi study;  questionnaire survey | Palliative and hospice  care specialty nurses | Enough knowledge | hospice and palliative care basics knowledge |
|  |  |  |  | knowledge of terminal illness |
|  |  |  |  | knowledge about drugs commonly used in hospice and palliative care |
|  |  |  |  | knowledge of social psychology and ethics |
|  |  |  |  | religious beliefs and funeral folklore knowledge |
|  |  |  | Other competence | primary care |
|  |  |  |  | symptom management for patients at the end of their lives |
|  |  |  |  | psychological and spiritual care |
|  |  |  |  | death care |
|  |  |  |  | grief counseling |
|  |  |  | Personal characteristics | responsibility |
|  |  |  |  | love |
|  |  |  |  | affinity |
|  |  |  |  | stability |
|  |  |  |  | confidence |
|  |  |  |  | cope with stress |
|  |  |  |  | self-care |
|  |  |  |  | reflective awareness |
|  |  |  | Motivation | self-improvement |
|  |  |  |  | accomplishment |
|  |  |  |  | personal career development |
|  |  |  | Self- concept | professional quality |
|  |  |  |  | values |
|  |  |  |  | professional identity |
|  |  |  | Social role | interpersonal communication |
|  |  |  |  | team building and collaboration |
|  |  |  |  | death education |
|  |  |  |  | propaganda |
|  |  |  |  | training and teaching |
| Wang,  2020,  China | qualitative study | Hospice nurses | Spiritual Care | help patients reflect on their lives |
|  |  |  |  | respect patients' beliefs |
|  |  |  |  | company and communicate |
|  |  |  |  | deal with unfulfilled wishes |
|  |  |  |  | reframe interpersonal relationships |
|  |  |  | Anticipatory grief intervention | information support and help |
|  |  |  |  | encourage family members to discuss |
|  |  |  |  | guide participation in caregiving |
|  |  |  | Bereavement care | posthumous care |
|  |  |  |  | instruction in post-hospital affairs |
|  |  |  |  | warm-hearted farewell |
|  |  |  |  | grief counseling |
| Autelitano,  2021,  Italy | organizational  case study;  literature review | Specialist palliative care nurses | Clinical competences and  activities | global assessment and symptom management |
|  |  |  |  | communication and educational competences |
|  |  |  |  | interprofessional work |
|  |  |  | Training activities | organize and support all members of the team in the design and implement training projects with evidence-based characteristics. |
|  |  |  | Research activities | not specified in the article |
| Wu,  2021,  China | qualitative study | Hospice nurses | Assist patients to live with quality | control symptoms of somatic pain and discomfort |
|  |  |  |  | relieve psychological pain |
|  |  |  |  | give life support |
|  |  |  |  | physical comfort care |
|  |  |  |  | psychological comfort care |
|  |  |  |  | environmental comfort care |
|  |  |  |  | social comfort care |
|  |  |  |  | spiritual and comfortable care |
|  |  |  | Assist patients to die with dignity | respect for life |
|  |  |  |  | fulfill unfulfilled wishes |
|  |  |  |  | adhere to ethics and morality |
|  |  |  |  | warm-hearted farewell |
|  |  |  | Support for patients' families | information provision and assistance |
|  |  |  |  | grief counseling |
|  |  |  | Teamwork | create a good team atmosphere |
|  |  |  |  | maintain effective operation of a team |
| Haavisto, 2021, Finland | qualitative study | Nurses in primary care settings | Ethics and courage in action | ethics in action |
|  |  |  |  | courage in action |
|  |  |  | Support for the patient | providing comfortable environment |
|  |  |  |  | psychological support |
|  |  |  |  | spiritual support |
|  |  |  | Support for the family | preparing family to end-of-life care of the patient |
|  |  |  |  | guidance in patient care |
|  |  |  |  | supporting the family after the patient’s death |
|  |  |  | Care planning | coordinating the care |
|  |  |  |  | keeping the care plan updated |
|  |  |  |  | documentation of care |
|  |  |  | Physical care | symptom management |
|  |  |  |  | fundamental care |
| Hokka, 2021, Finland | qualitative study；  open-ended questionnaire | Registered nurses | Basic palliative care:  (1) Competence in managing the most common symptoms | assess the patient's symptoms and define the need for treatment |
|  |  |  |  | master of pharmacological and nonpharmacological methods of symptom management |
|  |  |  |  | implement symptom-relieving care |
|  |  |  |  | assess physical symptoms and define the need for treatment |
|  |  |  |  | manage basics of symptom |
|  |  |  |  | assess psychosocial symptoms and define the need for treatment |
|  |  |  | Basic palliative care:  (2) Competence in supporting the patient and her/his closest ones | identify the need for, and implement psychosocial support |
|  |  |  |  | support the closest ones in palliative care |
|  |  |  |  | maintain hope |
|  |  |  |  | provide psychological support |
|  |  |  |  | coordinate spiritual support |
|  |  |  |  | involve the closest ones in care |
|  |  |  |  | support the patient in palliative care |
|  |  |  |  | utilize multi-professional support |
|  |  |  | Basic palliative care:  (3) Competence in basics of holistic palliative care | understand concepts and guidelines of palliative care |
|  |  |  |  | basic nursing care as a part of palliative nursing |
|  |  |  |  | palliative care of different patient groups |
|  |  |  |  | assess the need for palliative care |
|  |  |  |  | holistic palliative nursing |
|  |  |  |  | address oral, skin, position, and mobility issues in palliative care |
|  |  |  |  | nutrition as a part of palliative nursing |
|  |  |  | Basic palliative care:  (4) Competence in encountering the patient and her/his closest one | encounters with persons during palliative nursing |
|  |  |  |  | presence as a part of palliative nursing |
|  |  |  |  | genuine and respectful encounter |
|  |  |  | Basic palliative care:  (5) Competence of pain management and nursing care of patients in pain | assess pain |
|  |  |  |  | pharmacological methods of pain management |
|  |  |  |  | implement pain management and care the patients in pain |
|  |  |  |  | nonpharmacological methods of pain management |
|  |  |  |  | basics of pain management |
|  |  |  | Basic palliative care:  (6) Competence in social interactions in palliative care | social interactions as a part of palliative nursing |
|  |  |  |  | sensitivity and empathy in social interaction |
|  |  |  |  | verbal communication |
|  |  |  |  | break bad news |
|  |  |  | Basic palliative care:  (7) Competence in pharmacological treatment | implement pharmacological treatment in palliative care |
|  |  |  |  | basics of pharmacological treatment |
|  |  |  |  | assess and anticipate the need for pharmacological treatment and evaluate its effectiveness in palliative care |
|  |  |  |  | knowledge and skills required for verification of medical competence |
|  |  |  | Basic palliative care:  (8) Competence in education and consulting | educate the patient and the closest one |
|  |  |  |  | consult skills |
|  |  |  |  | identify of the need for a consultation |
|  |  |  |  | guide the working community |
|  |  |  |  | perception of a student |
|  |  |  |  | provide consultative support for the members of the working community |
|  |  |  | Basic palliative care:  (9) Competence in setting goals of care and advanced care planning | documentation as a part of palliative nursing |
|  |  |  |  | adhere to goals of care |
|  |  |  |  | implement advanced care plans |
|  |  |  |  | apply collaboration when drafting care plans |
|  |  |  |  | set concepts of goals of care |
|  |  |  | Basic palliative care:  (10) Competence in multi-professional collaboration | multiprofessional collaboration in implementation of palliative care |
|  |  |  |  | collaborate between the nurse and physician |
|  |  |  |  | the nurse works as a liaison person between the patient and the physician |
|  |  |  | Basic palliative care:  (11) Competence in coordination of palliative care | coordinate palliative nursing and end-of-life care |
|  |  |  |  | integrate the third sector with patient care |
|  |  |  |  | network collaboration |
|  |  |  | Basic palliative care:  (12) Unhesitant attitude in care | implement nursing care unhesitantly |
|  |  |  |  | unhesitant attitude in encounters and presence |
|  |  |  |  | break the bad news unhesitantly |
|  |  |  |  | bringing along one's own expertise unhesitantly |
|  |  |  | Basic palliative care:  (13) Competence in care of an end-of-life patient and her/his closest ones | care for a dying patient |
|  |  |  |  | identify of approaching death |
|  |  |  |  | give up unnecessary nursing practices |
|  |  |  |  | care after death |
|  |  |  | Basic palliative care:  (14) Competence in strengthening  one's own competence and self-awareness | develop one's own competences |
|  |  |  |  | compassionate oneself in palliative care |
|  |  |  |  | identify one's own emotions |
|  |  |  | Basic palliative care:  (15) Ethical and juridical competence | patient's autonomy |
|  |  |  |  | ethical aspects of palliative nursing |
|  |  |  |  | professionality |
|  |  |  |  | advocacy in promoting the patient's matters |
|  |  |  |  | patient's rights |
|  |  |  |  | truthfulness |
|  |  |  | Basic palliative care:  (16) Cultural competence | knowledge of different cultures |
|  |  |  |  | multiculturality in the implementation of palliative nursing |
|  |  |  | Basic palliative care:  (17) Competence in existential questions | encounter death |
|  |  |  |  | help in existential suffering |
|  |  |  | Specialist palliative care:  (1) Competence in encountering the patient and her/his closest  one | encounters with persons during palliative nursing |
|  |  |  |  | presence as a part of palliative nursing |
|  |  |  |  | genuine and respectful encounter |
|  |  |  |  | encounters with children |
|  |  |  |  | confidence in constructive encounters |
|  |  |  |  | patient-based encounters |
|  |  |  | Specialist palliative care:  (2) Competence in pharmacological treatment | implement pharmacological treatment in palliative care |
|  |  |  |  | basics of pharmacological treatment |
|  |  |  |  | assess and anticipate the need for pharmacological treatment and evaluate its effectiveness in palliative care |
|  |  |  |  | knowledge and skills required for verification of medical competence |
|  |  |  |  | extensive expertise in pharmacological treatment |
|  |  |  | Specialist palliative care:  (3) Competence in setting goals of care and advanced care planning | documentation as a part of palliative nursing |
|  |  |  |  | adhere to goals of care |
|  |  |  |  | implement advanced care plans |
|  |  |  |  | apply collaboration when drafting care plans |
|  |  |  |  | set concepts of goals of care |
|  |  |  |  | advanced expertise in setting of care goals |
|  |  |  |  | participate as an expert in advanced care planning and setting goals of care |
|  |  |  | Specialist palliative care:  (4) Competence in care of an end-of-life patient and her/his closest ones | care for a dying patient |
|  |  |  |  | identify of approaching death |
|  |  |  |  | give up unnecessary nursing practices |
|  |  |  |  | care after death |
|  |  |  |  | address patient's convictions at the end of life and after death |
|  |  |  |  | support the closest ones after the patient's death |
|  |  |  |  | assess unnecessary nursing practices |
|  |  |  | Specialist palliative care:  (5) Ethical and juridical competence | patient's autonomy |
|  |  |  |  | ethical aspects of palliative nursing |
|  |  |  |  | professionality |
|  |  |  |  | advocacy in promoting the patient's matters |
|  |  |  |  | patient's rights |
|  |  |  |  | truthfulness |
|  |  |  |  | assess ethical issues and discuss them with the patient |
|  |  |  | Specialist palliative care:  (6) Competence in existential questions | encounter death |
|  |  |  |  | help in existential suffering |
|  |  |  |  | advanced expertise in dealing with death |
|  |  |  |  | address existential suffering |
|  |  |  | Specialist palliative care:  (7) Competence in maintaining expertise and taking care of own well-being at work | active self-development |
|  |  |  |  | postgraduate education |
|  |  |  |  | strong clinical know-how |
|  |  |  |  | autonomous decision-making and expertise |
|  |  |  |  | critical thinking and reflection |
|  |  |  |  | recognize one's own limits and accept support |
|  |  |  | Specialist palliative care:  (8) Advanced symptom management in nursing care of patients in palliative care | extensive know-how in symptom management |
|  |  |  |  | assess and manage advanced symptoms |
|  |  |  |  | palliative sedation and the issues related to it |
|  |  |  |  | special techniques for the management of symptoms |
|  |  |  |  | autonomous management of symptoms |
|  |  |  |  | acute situations in palliative care |
|  |  |  | Specialist palliative care:  (9) Teaching, development, and research competence in palliative care | educate palliative care |
|  |  |  |  | develop palliative care |
|  |  |  |  | research phenomena linked to palliative care |
|  |  |  | Specialist palliative care:  (10) Extensive competence in palliative nursing care of special groups | palliative care for different special groups |
|  |  |  |  | palliative care for children and adolescents |
|  |  |  |  | palliative care for mentally retarded persons |
|  |  |  |  | palliative care for lonely persons |
|  |  |  | Specialist palliative care:  (11) Competence in advanced support to patient in palliative care, and her/his closest ones | assess the need for social support in patients and their closest ones, along with the provision of support |
|  |  |  |  | provide support for grief work |
|  |  |  |  | advanced psychosocial support |
|  |  |  |  | specialized support for families with children |
|  |  |  |  | collaborate with the third sector |
|  |  |  | Specialist palliative care:  (12) Extensive competence in coordination of palliative care | coordinate the patient's care chain and ensure the continuity of patient's care |
|  |  |  |  | coordinate large networks and manage the collaboration of networks |
|  |  |  |  | end-of-life care at home |
|  |  |  |  | effects of the care environment on the patient |
|  |  |  | Specialist palliative care:  (13) Advanced competence in patient education and consultations | advanced patient education in different situations |
|  |  |  |  | strong competence in consultations |
|  |  |  |  | consultative support for different palliative care provision levels and health care settings |
|  |  |  | Specialist palliative care:  (14) Advanced competence in pain management and pain management nursing | extensive expertise in pain management |
|  |  |  |  | manage special techniques in pain management |
|  |  |  | Specialist palliative care:  (15) Special competence in palliative care | extensive expertise in palliative care as a part of nurses’ work |
|  |  |  |  | assess and anticipate the patient's needs in special situations and anticipate them in palliative nursing |
|  |  |  | Specialist palliative care:  (16) Competence in demanding social interactions | manage demanding social interaction situations |
|  |  |  |  | break bad news with an active approach |
| Ma,  2021,  China | qualitative study | Hospice care  specialized nurses | Professional care capability | basic clinical nursing competency |
|  |  |  |  | be able to deal with complex cases |
|  |  |  |  | ability in developing hospice and palliative service of characteristics |
|  |  |  | Communication competency | information provision |
|  |  |  |  | serious disease communication |
|  |  |  |  | death education |
|  |  |  | Teamwork | act as a coordinator and liaison in developing a consistent treatment plan with patients and their family members in hospice and palliative care |
|  |  |  | Professional identity and self-identity | identify with the beliefs behind hospice and palliative care |
|  |  |  |  | self-identity and strong mental capability |
|  |  |  | Human care capability | be caring, patient, attentive, empathetic, and respectful to patients and their family members |
|  |  |  | Family support and grief counseling | not specified in the article |
|  |  |  | Hospice and palliative care construction | have the awareness to create a physical and a warm, loving humanistic environment suitable for terminal patients |
|  |  |  | Leadership | be caring, patient, attentive, empathetic, and respectful to patients and their family members |
| Pei,  2021,  China | literature review；experts consultation； pretest of oncology nurses | Oncology nurses | Education, Collaboration and Professional Development | be able to voluntarily participate in continuing medical education to improve hospice and palliative service capacity |
|  |  |  |  | be able to provide hospice and palliative knowledge and education to the whole population in multiple ways |
|  |  |  |  | be able to assist patients, family members and caregivers in gaining access to hospice and palliative information resources |
|  |  |  |  | be able to participate and discuss in multi-disciplined regular seminars on hospice and palliative care cases and join decision-making |
|  |  |  |  | be able to work with other care providers to ensure a seamless transition between different care facilities and hospice and palliative care services |
|  |  |  |  | be familiar with cutting-edge advances and industry dynamics in the field of hospice specialty care |
|  |  |  |  | be able to apply the principles of evidence-based medicine to solve problems in hospice clinical practice |
|  |  |  |  | be able to explore research opportunities in hospice clinical practice and implement them successfully |
|  |  |  |  | be ability to apply hospice research findings to nursing education and clinical practice |
|  |  |  |  | be able to advocate for the development and improvement of health and social policies related to hospice care at the appropriate level |
|  |  |  | Symptom control ability | be able to perform a comprehensive assessment of common symptoms (e.g., pain, malnutrition, etc.) in patients with terminal cancer and be able to differentiate |
|  |  |  |  | be able to grasp the key points of care for terminal cancer patients with common symptoms (e.g. pain), expected symptoms (e.g. chemotherapy-expected vomiting), and terminal emergencies (e.g. pleural effusion) |
|  |  |  |  | be able to explain the properties and usage of commonly used symptom control medications and instruct patients on the correct use of medications |
|  |  |  |  | be able to explain the role of adjuvant medications in symptom control |
|  |  |  |  | be able to identify potential side effects, interactions and complications of commonly used symptom control medications and implement effective preventive, observational and nursing measures |
|  |  |  |  | be able to properly evaluate the effect of symptom control and record it timely to provide a basis for the selection and adjustment of symptom control drugs |
|  |  |  |  | be able to recognize potential problems associated with the safety of symptom control medication management |
|  |  |  | Morality, ethics, and law | identify with the objectives, social and personal values of the hospice practice they are engaged in |
|  |  |  |  | be able to respect patients and family members preferences and their care decisions |
|  |  |  |  | be able to assist multidisciplinary teams in addressing common ethical issues for patients with terminal cancer |
|  |  |  |  | be able to apply ethical principles to the process of end-of-life care |
|  |  |  |  | be able to assist individuals and family members in exploring and resolving sensitive issues (e.g., sexuality, body image damage, etc.) |
|  |  |  |  | be able to practice according to the hospice clinical practice guidelines and management norms issued by health department |
|  |  |  |  | be able to educate individuals and family members on relevant potential legal issues |
|  |  |  | Communication and psychosocial support | be able to communicate effectively to facilitate discussion and make decision with family members |
|  |  |  |  | be able to tactfully communicate negative news to terminal cancer patients and families |
|  |  |  |  | provide professional psychological counseling and support to terminal cancer patients and their families timely |
|  |  |  |  | be able to assess the burden and stress associated with the primary caregiver and provide support to meet their needs |
|  |  |  |  | be able to develop stress coping skills to support coping with stress in the hospice care process |
|  |  |  | End-of-life care | be able to analyze the reasons behind discomfort in dying patients and actively take actions to provide comfort |
|  |  |  |  | recognize the signs of impending death in terminally ill patients, and understand the principles and methods of management |
|  |  |  |  | be able to support the needs of family members and organize dignified farewell and memorial ceremonies for terminally ill patients and their family members |
|  |  |  |  | be able to apply the principles and methods of death education to guide terminal cancer patients and their family members to face and accept death |
|  |  |  |  | understand the basics of religion and the understanding of death by patients of different religions |
|  |  |  |  | be able to understand local funeral customs and funeral culture |
|  |  |  | Bereavement care | be able to briefly describe the characteristics of the grief response of the bereaved |
|  |  |  |  | be able to distinguish the types and expressions of grief reactions of the bereaved |
|  |  |  |  | identify the stages of the bereaved person's grief response |
|  |  |  |  | be able to identify mourners in danger of grief |
|  |  |  |  | be able to grasp the key points of care for acute grief reactions of the bereaved |
| Suikkala, 2021, Finland | cross-sectional  qualitative study | Physicians and registered  Nurses (only nurses'  data were extracted in this table) | Palliative care competence at all  levels within health care and  social welfare services | palliative care competence across health settings |
|  |  |  |  | competence in home-based palliative care and end-of-life care |
|  |  |  |  | competence in working as a member of a multi-disciplinary team |
|  |  |  | Individualized palliative care  competence | competence to provide needs in outpatient palliative care |
|  |  |  |  | competence in providing equal palliative care for all patient groups |
|  |  |  |  | competence in dedicated palliative care in both malign and non-malign conditions and different patient groups |
|  |  |  |  | competence in patient- and family-centered palliative care |
|  |  |  |  | competence in symptom-based care |
|  |  |  |  | competence in advanced care planning and hospice care |
|  |  |  |  | competence in applying health technology |
|  |  |  |  | competence in respecting human dignity and self-determination within palliative care |
|  |  |  | Person-centered encounters  competence | respecting encounters with patients and their significant others |
|  |  |  |  | competence in delivering psychosocial support of patients and their significant others |
|  |  |  |  | competence in cultural and religious sensitivity |
|  |  |  | Systematic competence | competences based on changing palliative care needs and requirements. |
|  |  |  | Development in palliative care | continuing competence assurance |
|  |  |  |  | enhancing competence in palliative care through further and continuing education |
| Tian,  2021,  China | literature review；  qualitative study；  Delphi study；  optimal order  graph method | Hospice nursing  specialist nurses | Clinical care | hospice and palliative knowledge |
|  |  |  |  | pain assessment and management capabilities |
|  |  |  |  | symptom assessment and management capabilities |
|  |  |  |  | death education and counseling skills |
|  |  |  |  | physical care capability |
|  |  |  |  | disease observation capability |
|  |  |  | Psychological care | empathy |
|  |  |  |  | spiritual soothing ability |
|  |  |  |  | grief care capacity |
|  |  |  |  | emotional regulation and stress coping skills |
|  |  |  |  | communication skills |
|  |  |  |  | interpersonal relationships |
|  |  |  |  | Maintain the last dignity |
|  |  |  | Career development | education and consulting capabilities |
|  |  |  |  | research capability |
|  |  |  |  | self-learning capability |
|  |  |  |  | interdisciplinary teamwork skills |
|  |  |  | Supportive care | social and family support |
|  |  |  |  | spiritual support |
|  |  |  | Narrative Care | narrative care competency |
|  |  |  | Ethics and law | respect life |
|  |  |  |  | address ethical challenges in hospice and palliative care |
| White, 2021, Ireland | mixed-methods study | Nurses and healthcare assistants | Good multidisciplinary team communication | not specified in the article |
|  |  |  | Collaborate with members of the MDT to develop shared care planning | not specified in the article |
|  |  |  | Anxiety and fear symptoms care | not specified in the article |
| Zhao,  2021,  China | Delphi study | Palliative Care Advanced Practice  Nurse (APN) | Communication and cooperation competences | be able to communicate effectively with patients and their family members |
|  |  |  |  | be familiar with team members and be able to coordinate effectively |
|  |  |  |  | be able to act as a patient advocate for analysis, communication, negotiation, and resolution between all parties |
|  |  |  |  | be able to communicate and coordinate effectively in patient referrals to ensure continuity and homogeneity |
|  |  |  | Clinical practice competences | evaluate and manage the pain |
|  |  |  |  | family-based patient care in the dying phase |
|  |  |  |  | evaluate and manage common symptoms apart from pain and terminal acute illness |
|  |  |  |  | comfort care capability |
|  |  |  |  | be able to evaluate the impact of hospice and palliative care on patients and their family members |
|  |  |  |  | be able to assess the psychological and social support status of patients and families and provide appropriate guidance or referral |
|  |  |  |  | be able to assess and judge disease progression and the possible benefits and drawbacks of care |
|  |  |  | Professional development | introspection and active learning |
|  |  |  |  | self-evaluation and self-care |
|  |  |  |  | pushing the refinement of policy and procedure in the form of quality improvement through evidence-based and innovative thinking |
|  |  |  |  | research publication and other achievements |
|  |  |  |  | professional practice competences can be continually certified by professional organizations |
|  |  |  | Cultural and spiritual care competences | respect different culture and take care of patients from different cultural background |
|  |  |  |  | be able to assess the spiritual needs and provide proper care |
|  |  |  | Ethical and legal-related competences | be able to identify and respond to ethical dilemmas and legal issues, and communicate effectively with patients and families |
|  |  |  |  | be able to lead patients in developing pre-established medical care plans and communicate effectively |
|  |  |  |  | provide ethical instruction to other health professionals |
|  |  |  |  | be able to promote the establishment and improvement of ethical policies related to hospice and palliative care in the institution and promote the resolution of legal issues |
|  |  |  | Education competences | be able to adequately assess the needs and influencing factors of patients and families and provide health education |
|  |  |  |  | be able to apply appropriate teaching methods in health professional training |
|  |  |  |  | be able to provide hospice and palliative education to the public |
|  |  |  | System resource management capabilities | be able to prioritize quality improvement projects |
|  |  |  |  | be able to evaluate the quality of hospice and palliate and identify problems in scientific method |
|  |  |  |  | be able to lead and develop hospice and palliative quality improvement projects |
|  |  |  |  | be able to identify, manage, and utilize system resource effectively |
|  |  |  | Evidence-based nursing practice and research ability | be able to apply the best evidence in palliative care practice  be able to conduct the research fulfilling clinical needs |
| A framework for nurses working in  specialist palliative care,  2002,  United Kingdom | National or association framework | Level 1: Support worker or health care assistant (competence under this level were not extracted because of failed meeting the inclusion criteria) | NA | NA |
|  |  | Level 2: Qualified nurse | Communication skills | Knowledge 1: Aware of skills, interactions and theoretical models that underpin effective communication in palliative care. |
|  |  |  |  | Knowledge 2: Understands the impact that the health care assistant’s communication and approach may have on the wellbeing of the patient and care. |
|  |  |  |  | Knowledge 3: Knows the principles and aims of caring for patients with complex needs for example, psychological issues of anger, denial etc. |
|  |  |  |  | Knowledge 4: Aware of the main local, regional and national support groups. |
|  |  |  |  | Knowledge 5: Knows about patient or user initiatives. |
|  |  |  |  | Competences skills 1: Assesses, plans, implements, evaluates  and documents the care of patients with a range of problems or issues. |
|  |  |  |  | Competences skills 2: Recognizes the opportunity, by picking  up cues, to hold deeper discussions relating to psychological, emotional or spiritual issues. |
|  |  |  |  | Competences skills 3: Confidently facilitates and manages  interactions with patients and families. |
|  |  |  |  | Competences skills 4: Contributes to multidisciplinary planning and decision-making meetings. |
|  |  |  |  | Behaviour 1: Assertive |
|  |  |  |  | Behaviour 2: Uses initiative |
|  |  |  |  | Behaviour 3: Confident |
|  |  |  |  | Behaviour 4: Self-aware |
|  |  |  | Quality assurance | Knowledge 1: Familiar with the Nursing and Midwifery Council’s (NMC) Code of professional conduct and Code of practice. |
|  |  |  |  | Knowledge 2: Understands the principles of clinical governance and quality assurance. |
|  |  |  |  | Knowledge 3: Recognises the need for reflection to maintain standards and uses the process of significant event analysis. |
|  |  |  |  | Knowledge 4: Recognises the need for continuing professional development and evidence-based practice. |
|  |  |  |  | Knowledge 5: Aware of the impact of an ineffectual use of resources. |
|  |  |  |  | Competences skills 1: Identifies unsafe practice and responds appropriately. |
|  |  |  |  | Competences skills 2: Undertakes risk assessments. |
|  |  |  |  | Competences skills 3: Assesses own performance against  minimum standards. |
|  |  |  |  | Competences skills 4: Actively contributes to programme of  clinical audit. |
|  |  |  |  | Competences skills 5: Recognises own and others’ limitations. |
|  |  |  |  | Competences skills 6: Manages resources effectively. |
|  |  |  |  | Competences skills 7: Accesses and uses evidence-based practice. |
|  |  |  |  | Behaviour 1: Exercises judgement |
|  |  |  |  | Behaviour 2: Committed |
|  |  |  |  | Behaviour 3: Accountable |
|  |  |  |  | Behaviour 4: Confident |
|  |  |  | Clinical practice, job knowledge and skill | Knowledge 1: Knows about the principles and practice of palliative and specialist nursing care and symptom control. |
|  |  |  |  | Knowledge 2: Understands the psychological issues and family dynamics affecting terminally ill people and their carers and integrates this into practice. |
|  |  |  |  | Knowledge 3: Recognises the need for multidisciplinary teamwork in palliative and specialist care. |
|  |  |  |  | Competences skills 1: Holistically assesses psychological, cultural, social, legal, and ethical issues affecting the patient and their career’s well-being, care and treatment. |
|  |  |  |  | Competences skills 2: Applies appropriate clinical judgement,  in consultation with others, to provide nursing care that meets the complexity of the patient’s illness. |
|  |  |  |  | Competences skills 3: Understands the wide-ranging implications of the decision-making of care and treatment on the patient and career. |
|  |  |  |  | Competences skills 4: Evaluates outcomes of care and makes  alterations appropriate to the everchanging clinical situation. |
|  |  |  |  | Competences skills 5: Interprets basic clinical data to inform  decision-making. |
|  |  |  |  | Behaviour 1: Understanding |
|  |  |  |  | Behaviour 2: Responsive |
|  |  |  |  | Behaviour 3: Confident |
|  |  |  | Education | Knowledge 1: Aware of how to contribute to creating a positive learning environment. |
|  |  |  |  | Knowledge 2: Aware of different learning theories and their application. |
|  |  |  |  | Competences skills 1: Utilises both formal and informal teaching methods. |
|  |  |  |  | Competences skills 2: Uses creative and reflective skills with  other colleagues. |
|  |  |  |  | Behaviour 1: Confident |
|  |  |  |  | Behaviour 2: Self-aware |
|  |  |  |  | Behaviour 3: Facilitative |
|  |  |  |  | Behaviour 4: Knowledgeable |
|  |  |  | Management and leadership | Knowledge 1: Understands the hospice or unit’s clinical governance policy. |
|  |  |  |  | Knowledge 2: Recognises the importance of coordinating all aspects of the clinical area, including personnel. |
|  |  |  |  | Knowledge 3: Understands the importance of providing a specialist resource to internal and external agencies |
|  |  |  |  | Knowledge 4: Recognises the need for continuing professional development. |
|  |  |  |  | Competences skills 1: Describes the practical impact of clinical governance on patient care with the hospice. |
|  |  |  |  | Competences skills 2: Plans and organises effectively and is able to delegate. |
|  |  |  |  | Competences skills 3: Creates a clinical environment that is  conducive to staff support, learning and communication. |
|  |  |  |  | Competences skills 4: Understands the organisation’s complaints policy and what to do if there is an adverse incident involving patients, staff or drugs. |
|  |  |  |  | Competences skills 5: Provides relevant information and  makes appropriate referrals. |
|  |  |  |  | Competences skills 6: Reviews and critiques current specialist  palliative care educational material. |
|  |  |  |  | Competences skills 7: Identifies own boundaries and learning  needs. |
|  |  |  |  | Competences skills 8: Disseminates information on the specialty in the clinical area. |
|  |  |  |  | Competences skills 9: Is conversant with the concept of  reflective practice. |
|  |  |  |  | Competences skills 10: Understands the personal performance  review and development process. |
|  |  |  |  | Behaviour 1: Articulate |
|  |  |  |  | Behaviour 2: Motivated |
|  |  |  |  | Behaviour 3: Assertive |
|  |  |  |  | Behaviour 4: Demonstrates integrity |
|  |  |  |  | Behaviour 5: Fair |
|  |  |  | Research and development | Knowledge 1: Knows the principles of evidence-based practice. |
|  |  |  |  | Knowledge 2: Knows the clinical governance agenda. |
|  |  |  |  | Knowledge 3: Aware of the constraints and challenges associated with the limited research for palliative care practice. |
|  |  |  |  | Competences skills 1: Delivers competent palliative care, giving a rationale for decisions that is based on the available evidence. |
|  |  |  |  | Competences skills 2: Critically evaluates and reflects on practice that is founded upon clinical guidelines and protocols. |
|  |  |  |  | Competences skills 3: Promotes discussion and contributes to the development of clinical guidelines and protocols. |
|  |  |  |  | Competences skills 4: Seeks opportunities for clinical supervision to enhance the ability to reflect and improve upon practice. |
|  |  |  |  | Behaviour 1: Exercises judgement |
|  |  |  |  | Behaviour 2: Motivated |
|  |  |  |  | Behaviour 3: Interdependent |
|  |  |  |  | Behaviour 4: Confident |
|  |  |  | Grief, loss and bereavement | Knowledge 1: Recognises the needs of grieving people and acts appropriately. |
|  |  |  |  | Knowledge 2: Understands the theory of the grieving process. |
|  |  |  |  | Competences skills 1: Uses supportive listening skills to help  people adjust to their grief. |
|  |  |  |  | Competences skills 2: Responds to the needs of the individual who is bereaved, bringing assurance about the grief process. |
|  |  |  |  | Competences skills 3: Respects an individual’s beliefs, community, culture and religion. |
|  |  |  |  | Behaviour 1: Confident |
|  |  | Level 3: Senior qualified nurse | Communication skills | Knowledge 1: Knows about disease trajectories and treatment effects. |
|  |  |  |  | Knowledge 2: Understands the therapeutic nature of nursing and the impact of the nurse and patient relationship. |
|  |  |  |  | Knowledge 3: Knows about counselling approaches. |
|  |  |  |  | Knowledge 4: Understands the role of patient or user involvement in specialist palliative care. |
|  |  |  |  | Competences skills 1: Supports patients and families through uncertainty, using knowledge of the impact of disease and its  treatment to discuss care options and coping strategies. |
|  |  |  |  | Competences skills 2: Identifies and deals confidently with issues on the patient’s agenda that may potentially lead to psychological morbidity, referring to colleagues, as appropriate. |
|  |  |  |  | Competences skills 3: Creates an empowering and affirming  environment. Suggests a range of options and goals, whilst working with patients and relatives to make their own choices. |
|  |  |  |  | Behaviour 1: Creative |
|  |  |  |  | Behaviour 2: Influential in a one-to-one situation or a small group |
|  |  |  |  | Behaviour 3: Supportive |
|  |  |  | Quality assurance | Knowledge 1: Understands the professional and ethical issues surrounding decision-making, in both general terms and related specifically to palliative care. |
|  |  |  |  | Knowledge 2: Knows the principles of research and  evidence-based practice. |
|  |  |  |  | Competences skills 1: Utilises resources effectively and efficiently. |
|  |  |  |  | Competences skills 2: Contributes fully to the process and  outcome of clinical audit. |
|  |  |  |  | Competences skills 3: Facilitates the risk assessment process. |
|  |  |  |  | Competences skills 4: Critically analyses and develops safe  practice through policy development. |
|  |  |  |  | Competences skills 5: Initiates significant event analysis. |
|  |  |  |  | Behaviour 1: Innovative |
|  |  |  |  | Behaviour 2: Creative |
|  |  |  | Clinical practice, job knowledge and skill | Knowledge 1: Uses their knowledge of advanced illness, palliative care and oncology to inform a comprehensive assessment of the patient’s needs. |
|  |  |  |  | Knowledge 2: Understands the implications of complex clinical issues. |
|  |  |  |  | Knowledge 3: Understands the principles of informed and independent decision-making. |
|  |  |  |  | Knowledge 4: Knows about legal, ethical and professional nursing issues - such as informed choice, consent and  empowerment - in relation to nursing practice. |
|  |  |  |  | Competences skills 1: Critically assesses clinical situations  and interprets complex information. |
|  |  |  |  | Competences skills 2: Applies professional judgement to make  decisions and achieve appropriate care outcomes. |
|  |  |  |  | Competences skills 3: Develops empowering and facilitative  relationship with patients and carers to involve them in decision-making. |
|  |  |  |  | Competences skills 4: Acts as a prime resource in providing  advice, information and support to junior and other staff. |
|  |  |  |  | Competences skills 5: Applies knowledge of research principles and best practice to nursing care. |
|  |  |  |  | Competences skills 6: Identifies ideas and strategies that contribute to the development of care standards and processes. |
|  |  |  |  | Behaviour 1: A role model |
|  |  |  |  | Behaviour 2: Enquiring |
|  |  |  |  | Behaviour 3: Innovative |
|  |  |  |  | Behaviour 4: Dynamic |
|  |  |  | Education | Knowledge 1: Utilises in-depth knowledge of specialist palliative care and the way in which adults learn. |
|  |  |  |  | Knowledge 2: Knows about effective coaching and mentoring skills. |
|  |  |  |  | Competences skills 1: Articulates and reflects palliative care skills, enabling others to learn. |
|  |  |  |  | Competences skills 2: Facilitates and develops practice through clinical supervision. |
|  |  |  |  | Competences skills 3: Appreciates and refers to evidence to support practice. |
|  |  |  |  | Competences skills 4: Creates a positive learning environment. |
|  |  |  |  | Competences skills 5: Uses a range of teaching methods applicable to various situations. |
|  |  |  |  | Behaviour 1: Articulate |
|  |  |  |  | Behaviour 2: Supportive |
|  |  |  |  | Behaviour 3: Reflective |
|  |  |  | Management and leadership | Knowledge 1: Has a working knowledge of managerial and organisational theory, in order to provide leadership that is sensitive to the specialist palliative care environment. |
|  |  |  |  | Knowledge 2: Knows about change management and decision-making processes. |
|  |  |  |  | Knowledge 3: Understands the need to manage resources, within budgetary constraints. |
|  |  |  |  | Competences skills 1: Supports junior staff to develop skills  in organising, prioritising and delegating. |
|  |  |  |  | Competences skills 2: Ensures an appropriate induction or  development programme is in place. |
|  |  |  |  | Competences skills 3: Demonstrates effective management of  all complaints and incidents. |
|  |  |  |  | Competences skills 4: Identifies and manages poor performance. |
|  |  |  |  | Competences skills 5: Participates in the unit’s recognised  role in providing specialist advice and support. |
|  |  |  |  | Competences skills 6: Utilises resources effectively, organising appropriate cover for a clinical area that recognises the needs of specialist palliative care. |
|  |  |  |  | Competences skills 7: Develops individual learning outcomes  for clinicians. |
|  |  |  |  | Behaviour 1: Innovative |
|  |  |  |  | Behaviour 2: Sensitive |
|  |  |  |  | Behaviour 3: Enquiring |
|  |  |  |  | Behaviour 4: Confident |
|  |  |  | Research and development | Knowledge 1: Knows about the research process and of the challenges of outcome orientated research methodology in a palliative care setting. |
|  |  |  |  | Knowledge 2: Understands the implications, limitations and ethical dilemmas that may arise from the nature of palliative care research. |
|  |  |  |  | Competences skills 1: Consistently refers to research findings to support practice decisions. |
|  |  |  |  | Competences skills 2: Uses well developed reflective skills to evaluate current practice. |
|  |  |  |  | Competences skills 3: Collects data from a range of sources to inform and shape own ideas. |
|  |  |  |  | Competences skills 4: Identifies sources to keep up-to-date with evidence-based practice. |
|  |  |  |  | Competences skills 5: Critically analyses research findings and literature. |
|  |  |  |  | Competences skills 6: Facilitates discussion on palliative care research and evidence-based practice. |
|  |  |  |  | Behaviour 1: Influential |
|  |  |  |  | Behaviour 2: Demonstrates initiative |
|  |  |  |  | Behaviour 3: Solution-orientated |
|  |  |  | Grief, loss and bereavement | Knowledge 1: Knows how to distinguish between normal and abnormal grief. |
|  |  |  |  | Knowledge 2: Understands the boundaries of support offered. |
|  |  |  |  | Competences skills 1: Uses advanced counselling skills to support and empower patients and carers experiencing loss. |
|  |  |  |  | Competences skills 2: Refers appropriately to other agencies and services. |
|  |  |  |  | Competences skills 3: Assesses any risks associated with the individual needs of the bereaved person. |
|  |  |  |  | Competences skills 4: Encourages reflective practice to validate and, where possible, improve upon current practice. |
|  |  |  |  | Behaviour 1: Assured |
|  |  | Level 4: Specialist nurse | Communication skills | Knowledge 1: Knows the theory and practice of therapeutic nursing relationships. |
|  |  |  |  | Knowledge 2: Knows counselling techniques. |
|  |  |  |  | Knowledge 3: Understands family dynamics, and theories and models for supporting families in crisis. |
|  |  |  |  | Competences skills 1: Analyses complex patient situations  and shares experiences and insights with others. |
|  |  |  |  | Competences skills 2: Guides and supports others to improve  communication skills amongst the team. |
|  |  |  |  | Competences skills 3: Teaches communication skills in formal settings to specialist and non-specialist staff. |
|  |  |  |  | Competences skills 4: Creates an environment that allows  nurses to develop therapeutic relationships and enables junior staff to share their views of patient issues in multidisciplinary meetings. |
|  |  |  |  | Behaviour 1: Influential |
|  |  |  |  | Behaviour 2: Exercises judgement |
|  |  |  | Quality assurance | Knowledge 1: Knows how to lead and develop clinical governance and quality assurance programmes, specifically  within specialist palliative care. |
|  |  |  |  | Knowledge 2: Aware of developments in policy and strategic initiatives at local, regional and national levels, particularly related to specialist palliative care. |
|  |  |  |  | Knowledge 3: Acts as a resource on different approaches to quality specialist palliative care service provision. |
|  |  |  |  | Competences skills 1: Effectively uses audit and outcomes to  influence practice at every strategic level. |
|  |  |  |  | Competences skills 2: Critically evaluates own and others’  performance, through reflection and analysis. |
|  |  |  |  | Competences skills 3: Seeks opportunities to lead and support quality initiatives at local, regional and national levels. |
|  |  |  |  | Competences skills 4: Demonstrates robust resource management. |
|  |  |  |  | Competences skills 5: Actively involved in business planning. |
|  |  |  |  | Behaviour 1: Influential |
|  |  |  |  | Behaviour 2: Assertive |
|  |  |  |  | Behaviour 3: Visionary |
|  |  |  |  | Behaviour 4: Demonstrates clarity |
|  |  |  | Clinical practice, job knowledge and skill | Knowledge 1: Uses their expertise in the principles and practices of specialist palliative care and advanced nursing practice. |
|  |  |  |  | Knowledge 2: Knows about developing therapeutic relationships with patients and their carers to assist their informed choices for care and treatment. |
|  |  |  |  | Knowledge 3: Understands the immediate and long-term impact and outcomes of decisions for patients and carers. |
|  |  |  |  | Knowledge 4: Uses research and audit to determine evidence of best practice as a rationale for prescribed care. |
|  |  |  |  | Competences skills 1: Critically analyses complex clinical data and information to inform diagnosis and decision-making. |
|  |  |  |  | Competences skills 2: Practices independently, seeking to determine positive outcomes for patients. |
|  |  |  |  | Competences skills 3: Develops detailed standards, protocols  and care strategies in specialist palliative nursing care. |
|  |  |  |  | Competences skills 4: Influences others through dissemination of knowledge and information. |
|  |  |  |  | Competences skills 5: Contributes fully to audit of clinical  practice. |
|  |  |  |  | Competences skills 6: Actively seeks new knowledge innovations and creates appropriate change. |
|  |  |  |  | Behaviour 1: Influential |
|  |  |  |  | Behaviour 2: A leader |
|  |  |  |  | Behaviour 3: A change agent |
|  |  |  |  | Behaviour 4: A researcher |
|  |  |  |  | Behaviour 5: Empowering |
|  |  |  |  | Behaviour 6: Visionary |
|  |  |  | Education | Knowledge 1: Utilises well-developed academic and specialist palliative care skills to teach adults in a range of situations. |
|  |  |  |  | Knowledge 2: Takes account of factors that may inhibit individual learning about specialist palliative care. |
|  |  |  |  | Competences skills 1: Utilises a variety of different approaches to education. |
|  |  |  |  | Competences skills 2: Consistently draws on research and literature to influence specialist and advanced nursing practice. |
|  |  |  |  | Competences skills 3: Reflects upon and learns from own practice. |
|  |  |  |  | Behaviour 1: Creative |
|  |  |  |  | Behaviour 2: Enquiring |
|  |  |  |  | Behaviour 3: Motivating |
|  |  |  |  | Behaviour 4: Empowering |
|  |  |  |  | Behaviour 5: Innovative |
|  |  |  | Management and leadership | Knowledge 1: Understands advanced nursing practice, specifically specialist palliative nursing. |
|  |  |  |  | Knowledge 2: Is politically aware of the issues affecting the development of specialist palliative care. |
|  |  |  |  | Knowledge 3: Has sound knowledge of change management theory and practice. |
|  |  |  |  | Knowledge 4: Knows how to establish the strategic direction of the service. |
|  |  |  |  | Knowledge 5: Reviews and negotiates resources to enhance practice. |
|  |  |  |  | Competences skills 1: Uses audit, evaluation and research to  influence practice at local, regional and national levels. |
|  |  |  |  | Competences skills 2: Identifies areas and produces proposals for service development. |
|  |  |  |  | Competences skills 3: Uses networking and influencing skills  to ensure the voice of the specialty is heard. |
|  |  |  |  | Competences skills 4: Collates and uses health data to inform  policy and health care practice. |
|  |  |  |  | Behaviour 1: Self-assured |
|  |  |  |  | Behaviour 2: Facilitative |
|  |  |  |  | Behaviour 3: Inspirational |
|  |  |  | Research and development | Knowledge 1: Understands methods to disseminate research findings. |
|  |  |  |  | Competences skills 1: Leads research, evaluating results to spearhead changes in practice. |
|  |  |  |  | Competences skills 2: Acts as a role model in the integration of evidence-based practice in palliative care, across a multidisciplinary perspective. |
|  |  |  |  | Competences skills 3: Uses leadership supervisory and facilitation skills to promote awareness of research. |
|  |  |  |  | Competences skills 4: Proactively disseminates and implements research findings. |
|  |  |  |  | Competences skills 5: Contributes to both regional and national agendas to improve the evidence base for specialist palliative care. |
|  |  |  |  | Behaviour 1: Creative |
|  |  |  |  | Behaviour 2: Achievement orientated |
|  |  |  |  | Behaviour 3: Capable of making an impact |
|  |  |  |  | Behaviour 4: Tenacious |
|  |  |  | Grief, loss and bereavement | Knowledge 1: Has in-depth knowledge of the theories of grief, loss and bereavement. |
|  |  |  |  | Knowledge 2: Has a sound understanding of abnormal grief reactions. |
|  |  |  |  | Knowledge 3: Has comprehensive knowledge of bereavement services. |
|  |  |  |  | Competences skills 1: Advises the bereaved person on future care options. |
|  |  |  |  | Competences skills 2: Acts as a resource for those providing bereavement care. |
|  |  |  |  | Competences skills 3: Collects relevant data to monitor outcomes of bereavement care. |
|  |  |  |  | Behaviour 1: Facilitative |
|  |  |  |  | Behaviour 2: Responsive |
| Competency Standards for Specialist Palliative Care  Nursing Practice,  2005,  Australia | National or association framework | Specialist palliative care nurses | Therapeutic Relationships | Sensitively establishes, maintains and adapts effective therapeutic partnerships with individuals with life-limiting illnesses, their caregivers and family according to individual needs, circumstances and preferences. |
|  |  |  |  | Demonstrates respect for uniqueness and individual autonomy, when responding to the individual’s experiences and response to dying and bereavement. |
|  |  |  |  | Negotiates mutually agreed goals of care within a therapeutic environment, and facilitates personcentred decision making to promote optimal outcomes for individuals with life limiting disease, their caregivers and family. |
|  |  |  |  | Recognises the effects of the intimate and intense nature of caring for individuals with a life limiting disease, their caregivers and family has on the self and other members of the team, and responds effectively. |
|  |  |  | Complex Supportive Care | Demonstrates advanced palliative care knowledge and skills in meeting the multiple, complex care needs of individuals with life-limiting illnesses, their caregivers and family, across the continuum of care including bereavement, and in the context of an interdisciplinary approach to care. |
|  |  |  | Collaborative Practice | Demonstrates advanced skills in collaborating with individuals, their caregivers and family, other nurses and members of the health care team to promote optimal palliative care outcomes. |
|  |  |  |  | Builds the capacity of nurses, other health team members and the wider community to understand and respond to complex palliative care health and support needs for individuals, their caregivers, and family. |
|  |  |  | Leadership | Actively participates in professional activities that promote the continuing development of quality palliative care. |
|  |  |  |  | Actively participates in policy and service development activities that contribute to the delivery of quality palliative care. |
|  |  |  |  | Applies an advanced understanding of contemporary legal, ethical and professional standards relevant to the provision of quality palliative care services in the delivery and development of palliative care services. |
|  |  |  | Improving Practice | Creates and sustains processes which support a positive culture of continuous critical inquiry in the provision of palliative care. |
|  |  |  |  | Demonstrates an ongoing, high level commitment to critical reflection and continuous professional development as a specialist palliative care nurse. |
| Canadian Hospice Palliative Care  Association Nursing Standards  Committee, 2009 | National or association framework | Hospice palliative care nurses | Quality of Living-Dying | The HPC nurse focuses on the quality of the experience of the person who is living with and dying from a life-limiting illness, as well as the experience of the family. |
|  |  |  |  | The HPC nurse practices with respect for the personal meanings, specific needs and hopes of the person who is living in the last phase of his/her life and his/her family |
|  |  |  | Comfort | The HPC nurse utilizes a knowledge-based, systematic, holistic, and evolving approach to address symptoms and issues specific to the living-dying experience. |
|  |  |  | Transitions | The HPC nurse provides care throughout multiple illness trajectories of life-limiting illnesses, which may occur over a short period of time (sudden death) or may be a longer process (exacerbations of chronic illness or recurrences of cancer). The HPC nurse supports the individual and his/her family through these transitions, the dying process and throughout the grief and bereavement processes. |
|  |  |  |  | The HPC nurse assists persons and families to access and navigate the health-care system. |
|  |  |  | Quality and Safety | The HPC nurse practices in accordance with legislation, policies, guidelines, and tools pertaining to assessment, information sharing, decision-making, advance care planning, pronouncement of death, after death care, and grief and bereavement support. |
|  |  |  | Leadership | The HPC nurse advocates for and promotes high quality and safe palliative care. |
|  |  |  |  | The HPC nurse advances HPC nursing through the generation and application of knowledge and research. |
|  |  |  |  | The HPC nurse is an essential team member of the interprofessional team and establishes collegial partnerships and contributes to the professional development of students, peers, colleagues and others through consultation, education, leadership, and mentorship. |
|  |  |  |  | The HPC nurse communicates and advances the distinct contribution of nursing to the interprofessional team. |
|  |  |  | Personal and Professional Growth | The HPC nurse recognizes the privileges and challenges of working with persons who are living-dying and their families. |
|  |  |  |  | The HPC nurse understands his/her own personal experience in response to suffering and death. |
|  |  |  |  | The HPC nurse recognizes his/her personal needs and practices self-care while experiencing multiple losses during the care of persons who are dying and their families. |
| Palliative Care Nurses New Zealand,  2014 | National or association framework | Registered nurses who work in non-specialist or generalist (primary) palliative care settings | Core palliative care competences for all registered nurse domain 1: Professional responsibility | Applies legal and ethical decision-making principles in planning and delivering palliative care for people with a life-limiting condition |
|  |  |  |  | Demonstrates a holistic model of care for those with a life-limiting condition, encompassing the Māori philosophy of health and wellbeing; |
|  |  |  |  | Understands the role of others in palliative care and the accountability of the registered nurse in planning and evaluating care |
|  |  |  |  | Understands the impact that a life-limiting condition has on a person, their family and wider community, and provides support to facilitate their own decision-making Demonstrates practice which promotes safe and independent care for the person with a life-limiting condition while maintaining their quality of life |
|  |  |  |  | Recognizes the cultural uniqueness of individuals and their families and demonstrates the provision of sensitive and culturally appropriate nursing care to those with a life-limiting illness Demonstrates the ability to gain cultural support and assistance from appropriate sources if/when needed |
|  |  |  | Core palliative care competences for all registered nurse domain 2: Management of nursing care | Applies knowledge of the pathophysiology of common symptoms to achieve effective symptom control, thereby improving the quality of life and reducing the burden for people with a life-limiting condition |
|  |  |  |  | Undertakes a comprehensive nursing assessment and delivers nursing care that incorporates all aspects of the person – physical, spiritual, emotional, and social. |
|  |  |  |  | Accurately documents a nursing assessment, management plan and evidence of ongoing evaluation of agreed patient goals and symptom control and demonstrates understanding of the importance of confidentiality |
|  |  |  |  | Provides adequate explanation and support to those with a life-limiting illness and their families, facilitating informed decision-making regarding proposed plan of care. Demonstrates an ability to access other support (as required) in order to meet the person and their family need for information/explanation |
|  |  |  |  | Demonstrates knowledge of common palliative care emergencies and is able to plan care so as to anticipate and limit distress for those with a life-limiting condition and their families/whanau. Understands the normal responses to grief and loss, which may be expressed in emotions that are confrontational |
|  |  |  |  | Determines the effectiveness of nursing care on clinical outcomes via regular and ongoing assessment of and with the person with a life-limiting condition and their family. |
|  |  |  |  | Provides health education appropriate to the needs of the health consumer and their family within a nursing framework which enables self-management |
|  |  |  |  | Uses reflective practice to identify areas for further learning, and documents needs and actions in a professional portfolio Demonstrates an awareness of current gaps in knowledge of palliative care, and takes responsibility to seek and use available resources to develop the skills required to care for people with a life-limiting condition and their family/whanau Accesses advice, assistance, debriefing and direction as necessary |
|  |  |  |  | Engages in self-care practices and encourages colleagues with similar activities to foster a caring environment that supports all levels of staff through challenging end-of-life situations. Maintains professional development in regard to advancing knowledge and skills in palliative care |
|  |  |  | Core palliative care competences for all registered domain 3: Interpersonal relationships | Demonstrates empathy and sensitivity in developing a therapeutic relationship with patients and their family/whanau |
|  |  |  |  | Actively encourages a partnership model of care for people living with a life limiting condition and their families, acknowledging, and clarifying their goals, priorities, and choices in care |
|  |  |  |  | Demonstrates effective communication skills collaborating with the interdisciplinary team in order to achieve the best possible outcome for the patient and family |
|  |  |  | Core palliative care competences for all registered nurse domain 4: Interpersonal relationships | Communicates and networks with the wider interdisciplinary team and activates a referral as/when required. Demonstrates collaboration in delivery of palliative care with other members of the interdisciplinary-disciplinary team |
|  |  |  |  | Recognizes when there is a need for specialist palliative care input, and is aware of how to access this support |
|  |  |  |  | Participates in quality improvement activities promoting the development of quality palliative care within their practice setting |
|  |  | Registered nurses who work in a specialist palliative care setting | Specialty palliative care competences for registered nursing domain 1: Professional  responsibility | Practices in accordance with legislative, professional, cultural and ethical standards for palliative care nursing |
|  |  |  |  | Engages in and contributes to the local, national and  international activities regarding palliative care thereby influencing the professional standing and development of palliative care nursing in New Zealand |
|  |  |  |  | Uses appropriate mechanisms for monitoring own  performance and competence |
|  |  |  | Specialty palliative care competences for registered nursing domain 2: Management of nursing care | Participates in the safe and effective nursing care of  people with a life-limiting condition who have complex palliative care needs |
|  |  |  | Specialty palliative care competences for registered nursing domain 3: Interpersonal relationships | Develops therapeutic relationships with health consumers to anticipate and meet their multiple care needs across the palliative care continuum |
|  |  |  |  | Initiates and ensures ongoing collaborative relationships with health consumers and colleagues |
|  |  |  | Specialty palliative care competences for registered nursing domain 4: Inter-professional health care and quality improvement | Contributes to quality improvement activities that improve the delivery of specialist palliative care |
|  |  |  |  | Practices from an evidence-based Framework and  contributes to the development of evidence-based practice |
|  |  |  |  | Provides advice and mentorship to colleagues and other health professionals involved in the delivery of palliative care |
| Hospice and Palliative Nurses Association,  2015, America | National or association framework | Registered nurse and the advanced practice re levels of practice (APN) | Primary palliative nursing skills | Understanding of the natural trajectory of illnesses and conditions and critical decision-making points within this process. |
|  |  |  |  | Management and treatment of serious or life-threatening conditions, including symptom management and end-of-life care, must be evidence-based. |
|  |  |  |  | Discussion of advance care planning, goals of care, issues of advanced disease, and provision of psychosocial support for clients and their families of varying cultures. |
|  |  |  |  | Understanding of hospice and palliative care services, eligibility, and how to access these services in their setting and community. |
|  |  |  |  | Attention to population-specific concerns across the lifespan (e.g., pediatric oncology palliative care, geriatric oncology palliative care). |
|  |  |  | Specialty palliative nursing skills | Acquisition of knowledge about pathophysiology of diseases, pain and symptom management, counseling, and communication skills. |
|  |  |  |  | Possession of advanced knowledge about care of individuals with serious and/or life-threatening illness and individuals who are imminently dying. |
|  |  |  |  | Management of complex pain and symptoms using sophisticated regimens. |
|  |  |  |  | Utilization of expert communication skills for exploration of quality of life, illness understanding, promotion of informed decision-making, conflict negotiation or advanced disease. |
|  |  |  |  | Organization of a plan for a patient’s dying in terms of setting, proactive pain and symptom management, and education for patient, family and staff about the dying process. |
|  |  |  |  | Direction within transitions of care such as discharge into the community or admission into the hospital |
|  |  |  |  | Provision of psychosocial and emotional support to patients and family along the illness trajectory. Provision of consistent presence in the difficult journey. |
|  |  |  |  | Attention to cultural and spiritual dimensions of care as specified by the patient and family. |
